# Oxidative stress promotes cytotoxicity in human cancer cell lines exposed to *Escallonia spp.* extracts

**DOI:** 10.1186/s12906-024-04341-4

**Published:** 2024-01-13

**Authors:** Carlos Jara-Gutiérrez, Luis Mercado, Marilyn Paz-Araos, Carolyn Howard, Mario Parraga, Camila Escobar, Marco Mellado, Alejandro Madrid, Iván Montenegro, Paula Santana, Paola Murgas, Cristina Jimenez-Jara, Luis Guillermo González-Olivares, Manuel Ahumada, Joan Villena

**Affiliations:** 1 Centro de Investigaciones Biomédicas (CIB), Facultad de Medicina, Universidad de Valparaíso, Valparaíso, Chile; 2 Facultad de Medicina, Escuela de Kinesiología, Universidad de Valparaíso, Valparaíso, Chile; 3 Laboratorio de Genética e Inmunología Molecular, Instituto de Biología, Pontificia Universidad Católica de Valparaíso, Valparaíso, Chile; 4 Instituto de Investigación y Postgrado, Facultad de Ciencias de la Salud, Universidad Central de Chile, Santiago, 8330507 Chile; 5 Laboratorio de Productos Naturales y Síntesis Orgánica (LPNSO), Departamento de Ciencias y Geografía, Facultad de Ciencias Naturales y Exactas, Universidad de Playa Ancha, Avda. Leopoldo Carvallo 270, Playa Ancha, Valparaíso, 2340000 Chile; 6 Instituto de Ciencias Aplicadas, Facultad de Ingeniería, Universidad Autónoma de Chile, el Llano Subercaseaux 2801, San Miguel, Santiago, Chile; 7 Facultad de Medicina y Ciencia, Sede Patagonia, Universidad San Sebastián, Puerto Montt, Chile; 8 Doctorado en Ciencias e Ingeniería para la Salud, Universidad de Valparaíso, Valparaíso, Chile; 9 Academic Area of Chemistry, Universidad Autónoma del Estado de Hidalgo, Mineral de la Reforma, Hidalgo, Mexico; 10 Centro de Nanotecnología Aplicada, Facultad de Ciencias, Ingeniería y Tecnología, Universidad Mayor, Santiago, Chile; 11 Escuela de Biotecnología, Facultad de Ciencias, Ingeniería y Tecnología, Universidad Mayor, Santiago, Chile

**Keywords:** Selective oxycution, Cancer cell lines, Chilean native plants, Redox unbalance

## Abstract

**Background:**

Standard cancer treatments show a lack of selectivity that has led to the search for new strategies against cancer. The selective elimination of cancer cells modulating the redox environment, known as “selective oxycution”, has emerged as a viable alternative. This research focuses on characterizing the unexplored Escallonia genus plant extracts and evaluating their potential effects on cancer’s redox balance, cytotoxicity, and activation of death pathways.

**Methods:**

36 plant extracts were obtained from 4 different species of the Escallonia genus (*E. illinita* C. Presl, *E. rubra* (Ruiz & Pav.) Pers., *E. revoluta* (Ruiz & Pav.) Pers., and *E. pulverulenta* (Ruiz & Pav.) Pers.), which were posteriorly analyzed by their phytoconstituents, antioxidant capacity, and GC-MS. Further, redox balance assays (antioxidant enzymes, oxidative damage, and transcription factors) and cytotoxic effects (SRB, ∆Ψmt, and caspases actives) of those plant extracts were analyzed on four cell lines (HEK-293T, MCF-7, HT-29, and PC-3).

**Results:**

36 plant extracts were obtained, and their phytoconstituents and antioxidant capacity were established. Further, only six extracts had EC_50_ values < 10 µg*mL^− 1^, indicating high toxicity against the tested cells. From those, two plant extracts were selective against different cancer cell lines: the hexane extract of *E. pulverulenta*´s stem was selective for HT-29, and the ethyl acetate extract of *E. rubra*´s stem was selective for PC-3. Both extracts showed unbalanced redox effects and promoted selective cell death.

**Conclusions:**

This is the first study proving “selective oxycution” induced by Chilean native plant extracts.

**Supplementary Information:**

The online version contains supplementary material available at 10.1186/s12906-024-04341-4.

## Background

Reactive oxygen species (ROS) are a series of chemical species formed during oxygen´s cellular metabolism, which biologically participate in processes like homeostasis, defense, and signaling. However, these might become harmful to biomolecules, cells, and tissue [1] and even promote multiple pathologies (e.g., cancer) when a loss of the cellular redox balance occurs, a process known as oxidative stress, which can be enabled by external factors such as heavy metals, smoking, alcohol consumption, and UV radiation [2, 3]. In fact, among cancer hallmarks, high production of ROS is highlighted [4–7] as being linked to the initiation, promotion, and progression steps of this pathology [8]. Therefore, many potential cancer treatment therapies have focused on ROS concentration.

In 2011, Burgess introduced the term “selective oxycution,” which is defined as the selective killing of cancer cells by promoting a redox imbalance in cancer cells vs. nontumoral cells [9]. To accomplish it, four strategies have been proposed to inflict lethal damage or to trigger apoptosis in cancer cells: (1) direct exposure to ROS-generating agents (prooxidant agents); (2) inhibition of cancer cell´s antioxidant enzymes; (3) intracellular ROS production decrease (antioxidants), and (4) an appropriate combination of the three previous strategies [6, 10–13]. Within these strategies, several studies using natural compounds have shown selective killing in different cancer cell lines.

For instance, Trachootham et al. (2006) used β-phenyl ethyl isothiocyanate (PEITC), present in cruciferous plants, to effectively disable the glutathione antioxidant system and, therefore, generate ROS accumulation selectively in ovarian cancer cells [14]. Posteriorly, Juan et al. (2008) used resveratrol, a polyphenol found in black grapes and other fruits with a high antioxidant capacity, in colorectal cancer cells, seeing that it plays a significant role in reducing cell proliferation and promoting intrinsic apoptosis mechanisms [15]. These encourage the search for new anticancer natural compounds, especially looking into endemic plants.

In Chile and Argentina, Mapuche people are characterized by a deep knowledge of nature, where they give medicinal uses to native plants, with more than 700 medicinal native Mapuche plants identified. However, little is known about their content and biological activity [16]. Among these, plants belonging to the Escallonia genus have been less explored. Particularly, Barraco (*Escallonia illinita* Presl.; Saxifragaceae) is a key representative of the genus, which has been well characterized and recommended by folk medicine for the treatment of hepatic, venereal, renal, and respiratory diseases and infected wounds. However, there is a lack of scientific evidence that supports such benefits. Nonetheless, some anticancer potential has been described [17–20]. Further, there are three other species from the same genus (*E. rubra*, *E. revoluta*, and *E. pulverulenta*), of which there is no single study or evidence of their potential uses in biomedicine. Therefore, this work uses extracts from Escallonia genus plants to evaluate their potential “selective oxycution” on cancer cell lines of the colon, breast, and prostate by measuring their antioxidant and cytotoxic capacities and their effect on redox balance.

## Materials and methods

### Escallonia genus plant collection

*E. illinita, E. rubra, E. revoluta*, and *E. pulverulenta* were collected from Laguna Verde (Latitude: -33.1; Longitude: -71.6833) and Fundo Santa Ana (Latitude: -33.2167; Longitude: -71.4) at 460 m.a.s.l. in January 2019. Employed Escallonia specimens are cataloged as common species, and their extraction and usage were performed following Chilean legislation law 20,283 “about native forest recovery and forestry foment” (Ley 20,283 “Ley sobre recuperación del bosque nativo y fomento forestal”) and Decree 28 “rules about resources destined to native forest research” (Decreto 28 “que reglamenta los recursos destinados a la investigación del bosque nativo”) of the Ministry of Agriculture of Chile. All collected plants were identified by Patricio Novoa, Forest Engineer, Botanical Expert, and Chief of the Horticulture Department, “*Jardín Botánico Nacional*,” Viña del Mar, Valparaíso, Chile, considering the plant’s morphological properties. Vouchers for each specimen: Ei (VALPLA 2017-11), Er (VALPLO 2017-12), Ep (VALPLO 2017-13), Ere (VALPLO 2017-14), are kept at the CIFAR, Farmacopea Chilena, Universidad de Valparaíso, Valparaíso, Chile.

### Extraction procedure

The extraction of aerial parts from Escallonia specimens was performed by applying a non-lethal procedure. Selective pruning was done using the ANASAC PASTA PODA TPN-50 product and fungicide paint for pruning, and it involved different types of wounds on the plant. Each plant´s stem, leaves, and flowers were selected as the sections to obtain the extracts. To accomplish this, they were air-dried at room temperature and then subjected to successive extractions using different solvents of increasing polarity, like a previous report by Jara et al. (2017) [21]. Briefly, 300 g of dried plant material was added to 500 mL of each solvent (n-hexane (H), di-chloromethane (D), ethyl acetate (A), and ethanol (E)); then, the extraction of *E. illinita*, *E. rubra*, *E. revoluta*, and *E. pulverulenta* was completed in 48 h and replicated three times. All the obtained extracts were sonicated, concentrated in a rotary evaporator at 40 °C, and stored at room temperature in darkness.

### Phytoconstituent compounds and antioxidant capacity analysis of extracts

#### Total phenolic content determination

The amount of total phenolic compounds in the extracts was determined using the method reported by Waterman et al. (1994) [22], with minor modifications determined by our research team [21]. Each extracted sample (2.0 mg) was dissolved in 2.0 mL of ethanol. Then, 500 µL of the extracts were mixed with Folin-Ciocalteau reagent (2.5 mL, 0.2 N) and incubated for 5 min. Posteriorly, a 7.5% w/v Na_2_CO_3_ solution (2.0 mL) was added and incubated in darkness at room temperature for 2 h. Absorbance was measured in a spectrophotometer (RayLEIGH, UV-2601, China) at 700 nm using ethanol as the blank. Absorbance values obtained were interpolated using a Gallic acid standard curve (0–200 mg*L^− 1^), and the total phenolic content was expressed as mM of Gallic acid equivalents (mM GAE) per g of dried extract. All the measurements were carried out in triplicate.

#### Total flavonoid content estimation

The total flavonoid content was determined using the Dowd method, as adapted by Arvouet-Grand et al. (1994) [23]. For this, 1 mL of 2% w/v aluminum trichloride (AlCl_3_) in ethanol was mixed with the same volume of the extract’s solution in ethanol (1.0 mg*mL^− 1^). Then, the mixture was incubated for 10 min at room temperature, and absorbance was measured at 415 nm against a blank sample consisting of 1.0 mL extract solution with 1.0 mL of methanol without AlCl_3_. The absorbance values were interpolated using a quercetin calibration curve (0–100 mg*L-1). The total flavonoid content was expressed as mM of quercetin equivalents (mM QE) per g of dry extract. All the measurements were carried out in triplicate.

#### Total anthraquinones content estimation

This estimation was carried out using the protocol of Arvouet-Grand et al., adapted by Mellado et al. (2012) [24]. For this, a protocol like the previously exposed in Sect. 2.3.2 was followed, measuring absorbance at 486 nm. The absorbance values were interpolated using an emodin calibration curve (0–70 mg*L^− 1^). The total anthraquinones content was expressed as mM of emodin equivalents (mM EE) per g of dry extract. All the measurements were carried out in triplicate.

#### Total reactive antioxidant power (TRAP) assay

The method developed by Romay et al. (1996) [25] was slightly modified for this experiment. Briefly, a 10 mM solution of 2,2′-azo-bis(2-amidino propane) (ABAP) was mixed with the same volume of 150 µM solution of 2,2′-azinobi(3-ethylbenzothiazoline-6-sulphonic acid) (ABTS) using PBS 100 mM at pH of 7.4 (TRAP solution). The mixture was incubated at 45 °C for 30 min and then cooled to room temperature. Sample solution (10 µL, 1.0 mg*mL^− 1^ of each extract) was mixed with TRAP solution (990 µL), and the absorbance was measured after 50 s at 734 nm against the ABTS solution as the blank. The absorbance values were interpolated in a Trolox standard curve (0–120 mg*L^− 1^), and the TRAP values were expressed in mM Trolox equivalent antioxidant capacity (mM TEAC). All the measurements were replicated three times.

#### Radical scavenging assays using DPPH●

The DPPH assay was performed as described by Brand-Williams et al. (1995) [26] with minor modifications. The sample (100 µL, extracts at 0–10 mg*mL^− 1^) was mixed with a 50 µM DPPH● solution (2.9 mL) freshly prepared in ethanol. A 50 µM DPPH● solution (2.9 mL) with ethanol (0.1 mL) was used as the control. The samples and control solutions were incubated for 15 min at room temperature, and the absorbance was measured at 517 nm. The inhibition (%) was calculated employing the following equation:

1 $${\text{Inhibition}}\,\left( {\text{\% }} \right)\,{\text{=}}\,{\text{100}}\,{\text{\% }}\, \times \,{\text{((Acontrol}}\, - \,{\text{Asample)}}\,{\text{/}}\,{\text{Acontrol)}}$$

From the obtained Inhibition (%) values, the IC_50_ value was determined by the dose-response equation.

#### Hydrogen peroxide scavenging activity

The ability of extracts to scavenge hydrogen peroxide can be estimated according to the method described by Ruch et al. (1989) [27], with modifications. Hydrogen peroxide solution (40 mM) was prepared in 50 mM phosphate buffer (pH 7.4). Aliquots (0.1 mL) of different extracts were transferred into the test tubes, and their volumes were made up to 0.4 mL with phosphate buffer. After adding 0.6 mL hydrogen peroxide solution, tubes were vortexed, and after 10 min, the absorbance of the hydrogen peroxide was determined at 230 nm against a blank. The ability to scavenge hydrogen peroxide was calculated using the following equation:

2 $${{\text{H}}_{\text{2}}}{{\text{O}}_{\text{2}}}\,{\text{Inhibition}}\,\left( {\text{\% }} \right)\,{\text{=}}\,{\text{100}}\,{\text{\% }}\, \times \,{\text{((Acontrol}}\, - \,{\text{Asample)}}\,{\text{/}}\,{\text{Acontrol)}}$$

From the obtained Inhibition (%) values, the IC_50_ value was determined by the dose-response equation.

#### Ferric reducing antioxidant power (FRAP) assay

With slight modifications, ferric reducing power was measured as described by Dudonné et al. (2009) [28]. Freshly prepared TPTZ reactive (10 volumes of 300 mM acetate buffer, pH 3.6, with 1.0 volume of 10 mM TPTZ (2,4,6-tri(2-pyridyl)-s-triazine) in 40 mM hydrochloric acid, and 1.0 volume of 20 mM ferric chloride FRAP reagent (3.0 mL) was mixed with deionized water (300 µL) and the sample (100 µL, 1.0 mg*mL^− 1^ of each extract). The mix was incubated for 30 min at 37 °C in a water bath, and the absorbance was measured at 593 nm using ethanol as the blank solution. The obtained absorbance values were interpolated in a Trolox calibrate curve (0–200 mg*L^− 1^), and the FRAP values were expressed in mM Trolox equivalent antioxidant capacity (mM TEAC). All the measurements were performed in triplicate.

#### Chromatographic analysis

The n-hexane (H), dichloromethane (D), ethyl acetate (A), and ethanol (E) extracts were diluted with chloroform, and analysis by gas chromatography (Hewlett Packard, Palo Alto, CA, USA) was carried out according to the method detailed elsewhere [29, 30]. The operating conditions were as follows: on-column injection; injector temperature: 250 °C; detector temperature: 280 °C; carrier gas, He at 1.0 mL*min-1; oven temperature program: 40 °C increase to 260 °C at 4 °C *min-1, and then 260 °C for 5 min, to afford the best separation through a capillary Rtx-5MS column. Mass detector ionization employed an electron impact of 70 eV. Compounds in the chromatograms were identified by comparison of their mass spectra with those in the NIST/EPA/NIH Mass Spectral Library [31], following previous indications [32]. The retention indices were determined under the same operating conditions about a homologous n-alkanes series (C8–C36) by the equation:

3 $${\text{RI}}\,{\text{=}}\,{\text{100}}\, \times \,\left( {{\text{n}}\,{\text{+}}\,{\text{Tr}}\,\left( {{\text{unknown}}} \right)\, - \,{\text{Tr}}\,\left( {\text{n}} \right)\,{\text{/}}\,{\text{Tr}}\,\left( {\text{N}} \right)\, - \,{\text{Tr}}\,\left( {\text{n}} \right)} \right)$$

where n = the number of carbon atoms in the smaller n-alkane; N = the number of carbon atoms in the larger nalkane; and Tr = the retention time. Component relative concentrations were obtained by peak area normalization.

### Cultured cell lines

The following experimental established cell lines were obtained from the American Type Culture Collection (Rockville, MD, USA): MCF-7 (human breast cancer; ATCC NO. HTB-22), HT-29 (human colon cancer; ATCC NO. HTB-38), PC-3 (human prostate cancer ATCC NO. CRL-1435) and HEK-293T (human embryonic kidney ATCC NO. CRL-3216). All cell lines were grown in a Dulbecco´s Modified Eagle Medium/Nutrient Mixture F-12 (DMEM-F12) containing 10% FCS, 100 U/mL penicillin, 100 µg*mL^− 1^ streptomycin, and 1 mM glutamine. Cells were seeded into 96-well microliter plates at 100 µL, with 3 × 10^3^ cells/well plating density. After a 24 h incubation at 37 °C (under a 5% humidified carbon dioxide ambient to allow cell attachment), cells were treated with different concentrations of extracts and incubated for 72 h under the same conditions. A stock solution of extracts was prepared in ethanol, and the final concentration of this solvent was kept constant at 1%. Control cultures received only 1% ethanol.

### In vitro cytotoxicity screening by using sulforhodamine B assay

Sulforhodamine B (SRB) assay was used following the method of Skehan et al. (1990) [33]. Cell density was determined using a microplate reader (wavelength 540 nm). Untreated cells were used as the negative control, while cells treated with doxorubicin (DOXO) were used as the positive control. In addition, all the samples were tested from 100 to 5 µg*mL^− 1^ (concentration of extracts) using ethanol as the carrier solvent. All the measurements were replicated three times. Finally, Sigma Plot software was used to calculate the EC_50_ value, and the selectivity index (SI) was calculated in the extracts with EC_50_ ≤ 10 µg*mL^− 1^. The selectivity of each extract in each cell line was analyzed by calculating the selectivity index (SI) as EC_50_ HEK-293T/ EC_50_ cancer cell line. If the values of SI were equal to or greater than 2, it is said that the extract is selective [34].

### Oxidative stress assays in cell lines

Cells were seeded at 5 × 10^5^ per well of 100 mm cell culture plates and incubated at 37 °C in a 5% humidified CO_2_ ambient plus 95% air mixture per 72 h. Each cell line was treated with one of the extracts per 24 h, after which cells were washed three times with PBS 1X and detached with a 0.25% trypsin/EDTA (HyClone) solution for two minutes at 37 °C. Cells were then placed in a complete medium to inhibit the trypsin. Cells were then collected in sterile 15 mL tubes and centrifuged at 300 g for 10 min. The cell pellet was resuspended in lysis buffer (0.022 M Na_2_HPO_4_, 0.088 M NaH_2_PO_4_) diluted 1:15 in Milli Q and sonicated at 35 watts for the different oxidative stress assays. The enzymatic activity and protein oxidation were normalized by total protein (mg) [35].

### Antioxidant defenses in cell lines exposed to extracts

#### Superoxide dismutase (SOD) activity

This assay was performed according to Beauchamp and Fridovich (1971) [36], which is based on reducing cytochrome c by the superoxide radical in a xanthine/xanthine oxidase system. Briefly, 5 µL of cell lysates from different treatments was mixed with solution A, composed of 0.5 mM xanthine and 20 µM cytochrome c dissolved in a phosphate buffer (0.1 mM EDTA, 50 mM Na_2_HPO_4_ and 50 mM NaH_2_PO_4_, pH 7.8) and a solution B containing xanthine oxidase and 0.1 mM EDTA (1:40). Enzymatic activity was detected at 550 nm in a spectrophotometer (RayLEIGH, UV-2601, China). The obtained absorbance values were interpolated in a SOD calibration curve (U enzyme) and normalized by protein mass (mg of protein). All the measurements were carried out in triplicate.

#### Catalase (CAT) activity

According to the methods described by Aebi (1984) [37], the activity of catalase was determined by spectrophotometrically measuring the loss of absorbance at 240 nm. of a reaction mixture consisting of 100 µL of 0.3 M H2O2 in 2.9 mL of phosphate buffer (50 mM Na2HPO4 and 50 mM NaH2PO4, pH 7.8) and 50 µL of cell lysates from different treatments. The measurement was performed for 90 s in a spectrophotometer (RayLEIGH, UV-2601, China). The obtained absorbance values were interpolated in a CAT calibration curve (U enzyme) and normalized by the protein mass (mg of protein). Each sample was analyzed in triplicate.

#### Reduced glutathione /oxidized glutathione (GSH/GSSG) ratio assay

GSH/GSS assay was performed as described in Rahman et al. (2006) [38]. The assay is based on the reaction of GSH with DTNB (Ellman’s reagent), which produces the TNB chromophore; the latter has its maximal absorbance at 412 nm and oxidized glutathione–TNB adduct (GS–TNB). Briefly, cell lysates from different treatments were mixed with 3 mL of cold buffer (NaCl 0.15 M, Na2HPO4 0.01, NaH2PO4 0.01, pH 7.4) and centrifuged at 3000G for 15 min at 4 °C. The clear supernatant was used for the total GSH assay. The supernatant (100 µL treated with 2 µL 2-vinyl pyridine) was incubated at 37 °C with 700 µL of KPE buffer (0.1 M potassium phosphate buffer with 5 mM EDTA disodium salt, pH 7.5), 60 µL of 280 µM NADPH and 60 µL 10 mM DTNB (5,5’-dithio-bis (2-nitrobenzoic acid)) for 10 min at 30 °C to oxidize all GSH to GSSG. GSSG was then reduced by adding 60 µL GSH reductase. The rate of TNB formation was followed at 412 nm and was proportional to the sum of GSH and GSSG present. The rate was compared with a standard curve of GSH in buffer and normalized by protein mass (mg of protein). All the measurements were carried out in triplicate.

#### Total reactive antioxidant power assay in cell lines (TRAPc)

The same method that TRAP for extracts was used for cell lysate. One volume of 10 mM solution of ABAP (2,2′-azo-bis(2-amidino propane) was mixed with the same volume of 150 µM solution of ABTS (2,2′-azinobi(3-ethylbenzothiazoline-6-sulphonic acid) using PBS 100 mM at pH of 7.4 (TRAP solution). The mixture was incubated at 45 °C for 30 min and then cooled to room temperature. Cell lysate (10 µL) was mixed with the TRAP solution (990 µL), and the absorbance was measured after 50 s at 734 nm against the ABTS solution as the blank. The absorbance values were interpolated in a Trolox standard curve (0–120 mg*L^− 1^), and the TRAP values were expressed in mM Trolox equivalent antioxidant capacity (mM TEAC). All the measurements were replicated three times.

#### Nrf2 and FOXO3a RT-qPCR

According to the manufacturer’s recommendations, total RNA was extracted using TRIzol® RNA Isolation Reagent (Ambion, Thermo FisherScientific, Waltham, MA, USA). Template cDNA was obtained by reverse transcription of 1 µg of total RNA using iScript™ Reverse Transcription Supermix (Biorad, CA, USA). Reaction mixtures were incubated at 25° C for 5 min, 46° C for 20 min, and 95° C for 1 min.

Relative quantification of gene expression levels for nuclear factor erythroid 2–related factor 2 (Nrf2) and transcription factor forkhead box O-3 a (FOXO3a) genes was carried out by real-time quantitative PCR (RT-qPCR) on CFX96 Touch™ Real-Time PCR system (Biorad, Hercules, CA, USA) using cDNA samples obtained as described before. For this purpose, SsoAdvanced Universal SYBR Green Supermix (Biorad, CA, USA) was used according to the manufacturer’s instructions. Specific primers were designed for the amplification of each gen. Nrf2-F: 5´CAACTACTCCCAGGTTGCCC-3´, Nrf2-R: 5´-AGTGACTGAAACGTAGCCGA-3´; Foxo3a-F: 5´-ACAAACGGCTCACTCTGTCC-3´, FOXO3a: 5´-GGATGGAGTTCTTCCAGCCG-3´. Gapdh-F: 5´-GAAGGTGAAGGTCGGAGTC-3´, Gapdh-R: 5´-GAAGATGGTGATGGGATTTC-3´. Comparative cycle threshold (Ct) values were obtained after the RT-PCR reaction was performed. All quantifications were normalized by the corresponding expression of glyceraldehyde-3-phosphate dehydrogenase (GAPDH) mRNA that served as the normalizer gene. The relative quantification was performed using the 2-△△Ct method. RT-qPCR reactions were performed at least in triplicate.

### Oxidative damage and ROS in cell lines exposed to extracts

#### Lipid peroxidation measurement

Malondialdehyde (MDA), the primary marker in lipid peroxidation, was measured using the thiobarbituric acid reactive substances (TBARS) assay according to Esterbauer et al. (1982) [39]. 1 mL of cell lysates from different treatments was treated with 30% (w/v) trichloroacetic acid (TCA) and centrifuged for 15 min at 3,000 RPM. Then, 1 mL of the supernatant was mixed with 0.67% (w/v) thiobarbituric acid (TBA). Samples were boiled for 20 min, and their absorbance spectrum was recorded at wavelengths between 400 and 600 nm using a UV–visible spectrophotometer (RayLEIGH, UV-2601, China). The concentration of the TBA-MDA adduct was determined by extrapolation from an MDA calibration curve. Each sample was analyzed in triplicate.

#### Protein carbonyl content assay

Based on the reaction of carbonyl groups generated by protein oxidation with 2-4-ditrophenylyidrazine, this assay was performed according to Levine et al. (1990) [40]. 10 µL of cell lysates from different treatments were treated with 20% (w/v) TCA on ice for 5 min and centrifuged for 15 min at 11,000 RPM. The pellet was suspended in 1 mL of 0.3% 2-4-ditrophenylyidrazine in 2 M HCl, vortexed, and left in darkness for 1 h, with periodic shaking. Then, 0.5 mL of 50% TCA was added while vortexing the sample, which was kept on ice for 5 min and centrifuged at 11,000 RPM for 5 min. The pellet was suspended in 1 mL ethanol: ethyl acetate (1:1), vortexed, and centrifuged at 11,000 RPM for 5 min. This procedure was repeated three times, and the pellet was later dried with N2 gas. After that, 2 mL of 6 M urea was added to the pellet, and the sample was incubated at 37 °C for 30 min. The reaction product was measured in a spectrophotometer (RayLEIGH, UV-2601, China) at 370 nm. The reaction product was measured at 370 nm. Each sample was analyzed in triplicate.

#### Measurement of reactive oxygen species (ROS) production by flow cytometry

Briefly, cells were treated with extracts (5, 10, and 25 µg*mL^− 1^) for 12 h. Untreated cells were used as the negative control, while cells treated with daunorubicin (DNR) 1 µM were used as the positive control. Intracellular ROS levels were visualized after incubation with 2’,7’-dichlorodihydro-fluorescein diacetate (DCFH_2_-DA) at a final concentration of 10 µM. The fluorescent dye was added for the last 30 min of the extract treatment period. After the incubation, cells were washed once in PBS, trypsinized, and centrifuged. The pellet was resuspended in PBS and examined immediately by flow cytometry [41].

### Apoptosis in cell lines exposed to extracts

#### Determination of mitochondrial potential (ΔΨmt) by flow cytometry

Rhodamine 123 (Rho123), a cationic voltage-sensitive probe that reversibly accumulates in mitochondria, was used to detect changes in mitochondrial membrane potential [42]. Cells were incubated with extracts (5, 10, and 25 µg*mL^− 1^) for 24 h. Untreated cells were used as the negative control, while cells treated with Carbonyl cya-nide-4-(trifluoromethoxy)phenylhydrazone (FCCP) 1 µM were used as the positive control. Subsequently, cells were stained with rhodamine 123 (1 µM) and incubated in darkness for 1 h at 37 °C. Then, the medium was removed, and cells were washed twice with PBS. Later, cells were trypsinized and collected by centrifugation (10 min at 1500G). The supernatant was discarded, and the cell pellets were resuspended in PBS and analyzed by flow cytometry using the FL1 filter. Results are expressed as a percentage of cells stained with Rho123.

#### Determination of caspases activation by flow cytometry

The activity of caspases was determined by using a fluorescent inhibitor of caspases tagged with fluorescein isothiocyanate, FITC-VAD-FMK. The CaspACE™ FITCVAD- FMK In Situ Marker was obtained from Promega. Briefly, cells were treated with extracts (5, 10, and 25 µg*mL^− 1^) for 48 h. Untreated cells were used as the negative control, while cells treated with daunorubicin (DNR) 1 µM were used as the positive control. Cells were incubated with CaspACE™ FITC-VAD-FMK in darkness for 20 min at room temperature. Then, the medium was removed, and cells were washed twice with PBS. Exposed cells were collected by trypsinization and centrifugation (10 min at 1500G). The supernatant was discarded, and cells were resuspended in PBS and analyzed by flow cytometry using the FL3 filter. Results are expressed as a percentage of cells stained with CaspACE™FITC-VADFMK [43].

### Statistical analysis

All data were reported as mean values ± standard deviation (SD). Due to non-parametric data, a Kruskal-Wallis ANOVA was used with a confidence level of 95% with STATISTICA 7.0 software.

## Results

The plant´s constituents extraction with increased polarity solvents (hexane (H), di-chloromethane (D), ethyl acetate (A), and ethanol (E)) resulted in 36 extracts, with 8 from *E. illinita* (4 from the stem and 4 from leaves), 8 from *E. rubra* (4 from the stem and 4 from leaves), 8 from *E. revoluta* (4 from the stem and 4 from leaves), and 12 from *E. pulverulenta* (4 from the stem, 4 from leaves and 4 from flowers).

### Phytochemical content and total antioxidant activity of extracts

Once the extracts were obtained, the phytochemical content (i.e., total phenolic contents, flavonoids, and anthraquinone) was measured using colorimetric assays, as summarized in Table S1. To highlight, total phenols and flavonoids showed significant differences in polar extracts (A, E) (*p* < 0.05) when compared to nonpolar solvents (H, D). Further, *E. pulverulenta* showed the highest phenolic content in the ethanol extract from the leaves (E = 0.7015 ± 0.0203 mM GAE) and flowers (E = 0.6461 ± 0.0057 mM GAE) organs. On the other hand, *E. illinita* had the highest content of flavonoids in the dichloromethane extract (D = 0.6189 ± 0.0352 mM QE) and the ethylacetate extract (A = 0.7032 ± 0.0001 mM QE), both from leaves. Finally, it was determined that the highest content in total anthraquinones was found in the hexane extracts (H = 0.0597 ± 0.0006 mM EE) and in the dichloromethane extracts (D = 0.0648 ± 0.0007 mM EE) made from leaves of *E. revoluta*, and in the hexane extracts (H = 0.0769 ± 0.0002 mM EE) and the dichloromethane extract (D = 0.0660 ± 0.0004 mM EE) of *E. rubra*´s stem and leaves, respectively.

The antioxidant activity of the extracts is summarized in Table S2. Interestingly, for the TRAP assay, all the extracts were less active than the positive controls (Gallic acid (GA) and Butylhydroxytoluene (BHT), *p* < 0.05). Yet, the ethanol extract from *E. pulverulenta*´s stem (E) shows the highest antioxidant capacity (*p* < 0,05). Similarly, the DPPH• assay showed that all ethanolic extracts show significantly higher values than the rest of the extracts (*p* < 0,05), especially in the ethanolic extract from the *E. pulverulenta*´s flower (E). However, none of the extracts had a better DPPH• scavenging capacity than reference antioxidant compounds (BHT and TROLOX®). In terms of the H_2_O_2_ assay, it was found that the dichloromethane extracts from *E. illinita*´s stem (D) showed a significantly higher H_2_O_2_ scavenger activity than the rest of the extracts (*p* < 0,05). This extract has an improved H_2_O_2_ scavenger capacity compared to the reference antioxidant compounds (BHT and TROLOX®). Finally, the FRAP assay showed that A and E extracts had better reduction antioxidant power than the other extracts (*p* < 0.05), where the highest value was found in the ethanolic extract from *E. pulverulenta*´s leaves (0.00076 ± 0.00001 mM TEAC). Nonetheless, all the extracts were less active than the positive controls (GA and BHT, *p* < 0.05). Therefore, it can be concluded that polar extracts have a higher concentration of phytoconstituents and higher total antioxidant capacity than the rest of the tested samples.

### In vitro cytotoxicity

The cytotoxicity of the thirty-six extracts was evaluated in vitro against different cancer cell lines (HT-29, PC-3, MCF- 7, and HEK-293T), using a sulforhodamine B colorimetric assay, which was set up to obtain the EC_50_ values of the tested extracts. Table 1 shows the results of extracts with EC_50_ values within the tested ranges.

**Table 1:** Effective mean concentration (EC_50_) of Escallonia extracts on nontumor (HEK-293T), breast cancer (MCF-7), colon cancer (HT-29), and prostate cancer (PC-3) cell lines

Plant	Organ	Extract	MCF-7 EC_50_ (µg*mL^− 1^)	HT-29 EC_50_ (µg*mL^− 1^)	PC-3 EC_50_ (µg*mL^− 1^)	HEK-293T EC_50_ (µg*mL^− 1^)
*E. illinita*	Stem	D	44.0401 ± 2.8211^c^	24.9665 ± 3.0838^c^	63.0176 ± 2.5672^d^	22.7509 ± 1.5763^c^
		A	13.5532 ± 4.4832^a^	34.6422 ± 4.7805^c^	71.3293 ± 3.7622^d^	18.6287 ± 4.4183^b^
*E. rubra*	Stem	H	10.1507 ± 1.5942^a^	9.3007 ± 2.9967^a^	8.2581 ± 3.1385^a^	13.9341 ± 1.9361^a^
		D	23.3823 ± 1.3818^b^	19.9153 ± 5.1146^b^	14.7452 ± 1.9173^b^	11.6367 ± 5.1136^a^
		A	12.7918 ± 0.9803^a^	26.375 ± 0.3326^c^	6.7183 ± 2.7161^a^	14.7107 ± 2.2366^a^
	Leaves	H	56.1876 ± 2.6042^c^	15.2167 ± 1.5197^d^	9.5785 ± 2.3736^a^	10.2952 ± 2.2208^a^
		D	16.7674 ± 2.6103^b^	25.0321 ± 6.5080^c^	14.0886 ± 1.1765^b^	11.3583 ± 2.5385^a^
*E. revoluta*	Stem	H	39.7867 ± 3.1283^c^	24.4083 ± 2.0075^c^	79.3285 ± 1.2329^d^	14.1480 ± 1.3285^a^
		D	18.3568 ± 1.8974^b^	13.7411 ± 2.1074^d^	14.7673 ± 4.1305^b^	10.6601 ± 1.9422^a^
		A	22.5015 ± 4.4417^b^	6.9845 ± 1.2959^a^	10.7772 ± 0.6380^a^	11.0185 ± 3.4961^a^
*E. pulverulenta*	Stem	H	17.7417 ± 1.5066^b^	7.5154 ± 1.9235^a^	11.2879 ± 3.2494^b^	17.3630 ± 8.3298^b^
		D	95.1946 ± 10.9158^d^	24.3763 ± 2.4381^c^	20.3552 ± 8.8824^c^	12.0393 ± 1.7299^a^
		A	24.0417 ± 2.5179^b^	23.9766 ± 1.0591^b^	23.0374 ± 4.3020^c^	11.8085 ± 1.8638^a^
	Leaves	H	20.3313 ± 1.6390^b^	19.458 ± 2.0729^b^	21.4674 ± 3.9661^c^	32.1562 ± 14.5701^d^
		D	55.4643 ± 7.1389^d^	13.6433 ± 1.9642^d^	22.6237 ± 4.5147^c^	13.9832 ± 0.8228^a^
		A	15.0914 ± 1.7535^b^	22.9409 ± 9.5515^b^	22.8511 ± 7.6715^c^	18.0282 ± 2.5183^b^
	Flowers	H	15.4196 ± 2.8012^a^	30.2962 ± 3.7342^c^	18.5459 ± 2.5133^c^	15.2301 ± 3.4980^b^
		D	9.3985 ± 1.8046^a^	9.3284 ± 3.7723^a^	8.6317 ± 2.9129^a^	13.8068 ± 2.7420^a^
		A	19.7591 ± 4.4834^b^	13.4092 ± 3.8624^d^	19.5506 ± 3.8179^c^	11.3231 ± 3.1435^a^
		E	15.9509 ± 2.2752^a^	14.7024 ± 0.9387^d^	20.5502 ± 4.7137^c^	15.2715 ± 3.3880^b^

Data are expressed as mean values ± S.D. (*n* = 10)

^a-d^Different letters correspond to significant differences among the extracts per cell line (*p* < 0.05)

From Table 1, it is possible to identify that the EC_50_ values ranged from 6.7 to 95.2 µg*mL^− 1^ for the different cancer cell lines tested. Therefore, to continue with the study, the 6 extracts with the lowest EC_50_ value (≤ 10 µg*mL^− 1^, highest cytotoxicity) were selected, and the selectivity index (SI) was calculated (Table S3).

Table S3 shows SI results for twenty Escallonia extracts, where only two extracts showed to act selectively against cancer cells; specifically, ethyl acetate extract from *E. rubra*´s stem (ErSA) was selective for prostate cancer cell line PC-3 (SI = 2.19) and hexane extract from *E. pulverulenta*´s stem (EpSH) was selective for colon cancer cell line HT-29 (SI = 2.31). Therefore, the research was followed, considering only these two extracts. Note that the SI values from the extracts were compared to the SI from doxorubicin (DOXO), a chemo-therapeutic used as a gold standard, which was selective for the three cancer cell lines, being their SI values for colon and breast cancer cell lines higher than any other SI from the extracts.

### Antioxidant defenses in cell lines exposed to extracts

The two selected extracts (ErSA and EpSH) were used to analyze their impact on the oxidative stress in the cell lines employed (HEK-293T, HT-29, and PC-3). In this sense, the oxidative stress post exposition to the extracts was evaluated through the analysis of intracellular antioxidant defenses (SOD and Catalase enzymes activity, GSH/GSSG ratio, TRAP in cell, Nrf2 and FOXO3a transcription factors) and oxidative damage (lipid peroxidation, carbonyl formation, and ROS production).

Related to the SOD and Catalase enzymatic activity (Figure S1), the ErSA extract was selective for the PC-3 cell line showing a significant increase in SOD activity (Figure S1c). On the other hand, ErSA and EpSH extracts were significantly more active for catalase on cancer cell lines than nontumor-treated cells (Figure S1 d, e, and f). Regarding the GSH/GSSG ratio, both extracts increased the ratio in HEK-293T, which indicates a reduction of oxidative stress (Figure S2a). Further, the analysis of the antioxidant capacity in cells (TRAPc) showed that both extracts diminished TRAPc in HEK-293T, indicating a prooxidant effect (Figure S2d). Also, the ErSA extract (selective for the PC-3 cell line) significantly reduced TRAPc, demonstrating a prooxidant effect (Figure S2f).

**Fig. 1:**
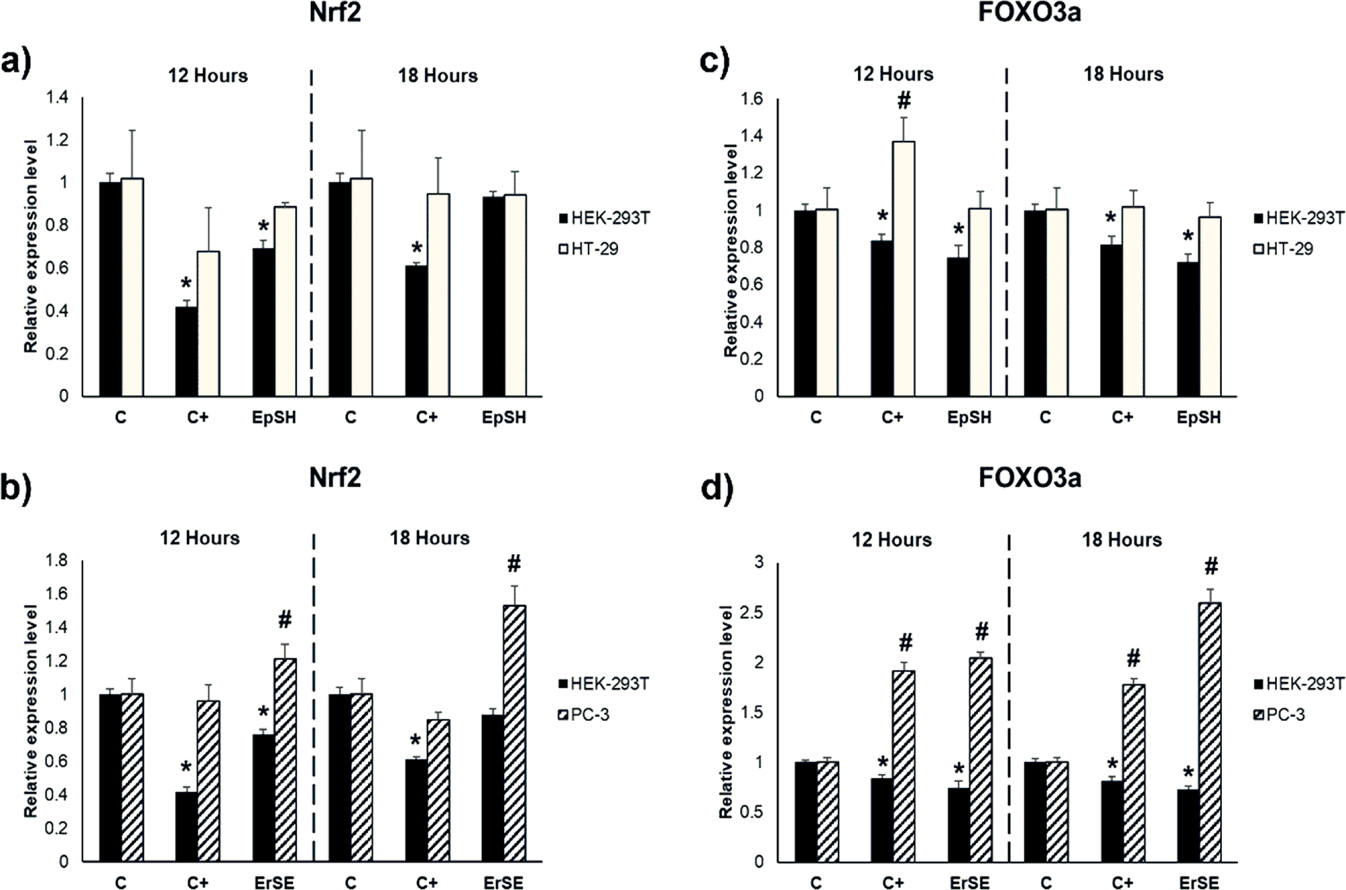
Relative expression of Nrf2 (**a**, **b**) and FOXO3a (**c**, **d**) in HEK-293T, HT-29, and PC-3 after treatment with ErSE and EpSH extracts. Cells were treated with the ErSE or EpsH extracts for 12 and 18 h (25 µg*mL^− 1^). Data were expressed as mean values ± standard deviation (*n* = 3). *# Different symbols correspond to significant differences between treatments concerning the negative control (C) per cell line (*p* < 0,05). C = Negative Control; ErSE = ethyl acetate extract made from stems of *E. rubra*, selective for PC-3; EpSH = hexane extract made from stems of *E. pulverulenta*, selective for HT-29; C + = 5-Fluorouracil (6.5 µg*mL^− 1^, 50 µM)

As enzymatic antioxidant defenses are increased, we studied the expression of transcription inducible factors, Nrf2 and FOXO3a, as regulator factors in the enzymatic response to ROS production (Fig. 1a-d). ErSA extract significantly increased the expression of both transcription factors in PC-3 cells (Fig. 1b and d), which is related to the increase in the activity of SOD and catalase in the same cell line (Figure S1c and f) and reduced the expression of both factors in HEK-293T control cells. Meanwhile, EpSH extract did not affect the expression of Nrf2 and FOXO3a in HT-29 colon cancer cells and reduced the expression of both transcription factors in HEK-293T control cells (Fig. 1a and c).

### Oxidative damage and ROS level in cell lines exposed to extracts

The oxidative damage in cell lines was evaluated by measuring lipid peroxidation and carbonyl concentration (Figure S3a-f). The ErSA extract significantly increased the concentration of carbonyls in the PC-3 cell line, while no change was observed in HEK-293T control cells. On the other hand, EpSH extract showed increased carbonyl concentration and lipid peroxidation in HT-29 cells while reducing the lipid peroxidation in HEK-293T control cells.

**Fig. 2:**
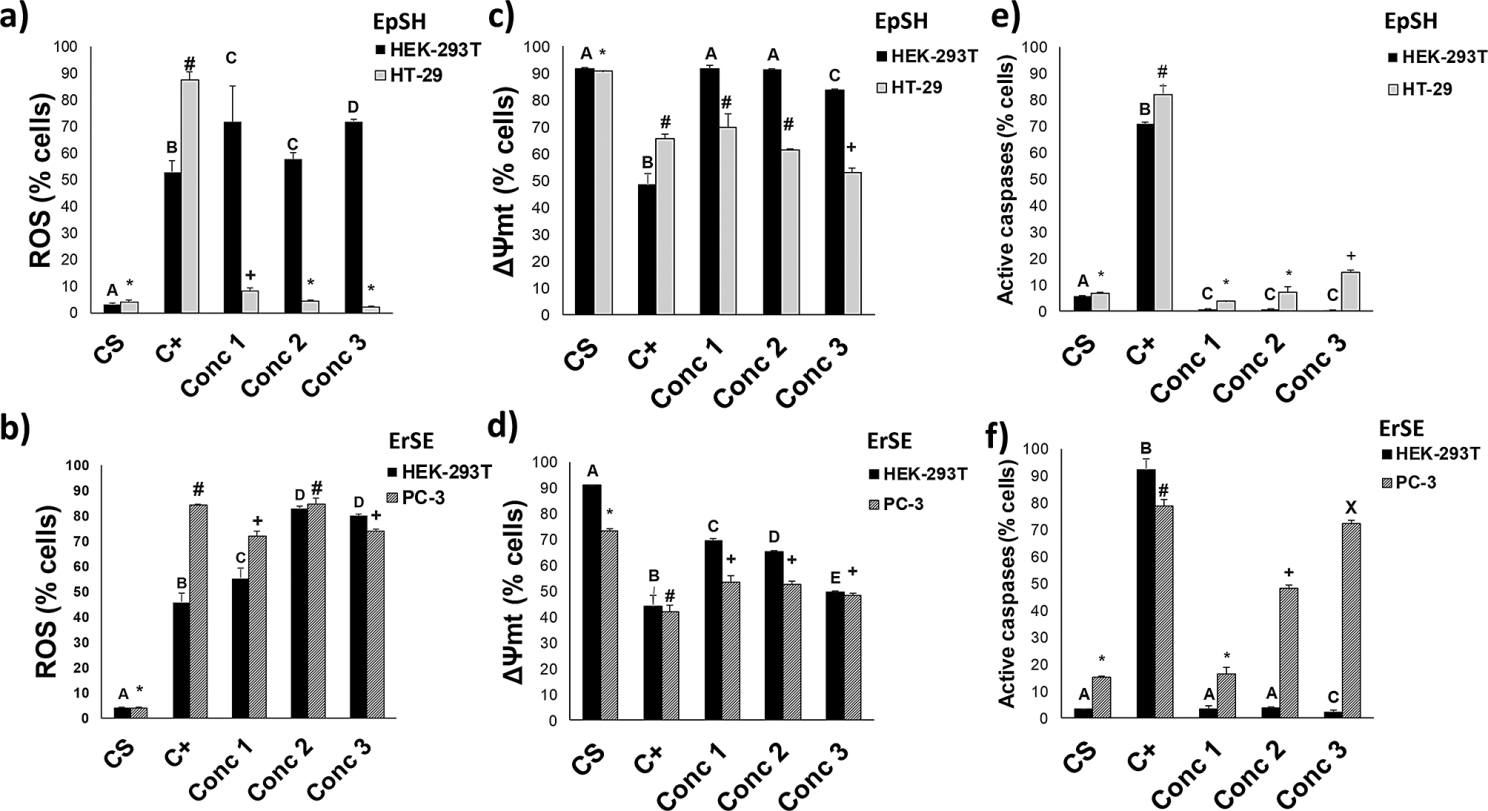
ROS production, loss of mitochondrial membrane potential (ΔΨmt), and caspases activity in nontumor (HEK-293T) and colon (HT-29) and prostate (PC-3) cancer cell lines after being exposed to three different concentrations (C1 = 5 µg*mL^− 1^, C2 = 10 µg*mL^− 1^, C3 = 25 µg*mL^− 1^) of EpSH and ErSA extracts, respectively. (**a**) and (**b**) Mean percentage of cells with ROS production. (**c**) and (**d**) Mean percentage of cells with ΔΨmt. (**e**) and (**f**) Mean percentage of cells with active caspases. For the ROS production, 1 µM daunorubicine (DRN) as a positive control (C+), for the mitochondrial membrane potential, 1 µM Carbonylcyanide-p-trifluoromethoxyphenylhydrazine (FCCP) as a positive control (C+), while 1 µM daunorubicin (DRN) was used as a positive control (C+) for caspase activation. The solvent control was 0.1% ethanol (CS) in all cases. ^A−E^ Different letters and ^*#+X^ symbols correspond to significant differences among treatments per cell line (*p* < 0.05)

Interestingly, when the ROS levels in the cells were evaluated after exposure to the extracts, it was found that both ErSA and EpSH increased the ROS levels in PC-3 and HT-29 cells, respectively, similarly to the positive control daunorubicin (DNR, 1 µM). Further, ErSA extract generated ROS in the PC-3 cells and the control cell in a concentration-dependent manner, like the reference chemo-therapeutic used as a positive control (DNR) (Figs. 2b and S4b). Nonetheless, for EpSH extract, the ROS level was significantly higher in HEK-293T than in HT-29 cells (Figs. 2a and S4a).

### Apoptosis in cell lines exposed to the extracts

It has been reported that cellular apoptosis activation could be associated with mitochondrial membrane potential and caspase activation through ROS levels [44]. Therefore, the effect of EpSH and ErSA extracts on mitochondrial membrane potential (ΔΨmt) and caspase activation on the tested cell lines was assessed. As shown in Figs. 2c, d and S4c, d, both extracts decreased the mitochondrial membrane potential in cancer cell lines (PC-3 and HT-29) in a dose-dependent manner, like the positive control. Furthermore, the ErSA extract also reduced the mitochondrial membrane potential at the highest concentration (25 µg*mL^− 1^) in non-tumoral HEK-293T cells, having no significant differences with PC-3 cells, suggesting a selective effect on the mitochondrial membrane potential. Related to the caspase activation, both extracts showed increased activation in the cancer cell lines, HT-29 and PC-3, indicating the apoptotic pathway activation (Figs. 2e, f, and S4e,f). Both extracts showed increased caspase activity in the cancer cell lines HT-29 and PC-3, indicating the activation of the apoptotic pathway. On the one hand, ErSA extract significantly increased the caspase activity of the PC-3 cancer cell line without any other effect on nontumor HEK-293T cells. On the other, the impact of EpSH extract significantly increased the caspases activity at the highest concentration on HT-29 cells, leaving HEK-293T cells unaffected. Thus, it was observed that both extracts also promote a selective effect in terms of caspase activity. Therefore, summarizing, ErSA extract promotes an increase in ROS levels, decreasing the mitochondrial membrane potential and increasing caspase activity in PC-3 cells, showing a selective effect in caspase activation versus nontumor HEK-293T cells. Likewise, the EpSH extract increases the ROS levels, decreasing the mitochondrial membrane potential and increasing the caspase activity in HT-29 cells, where no similar effects were observed in the nontumor cell line, thus demonstrating a selective effect on HT-29 versus HEK-293T cells.

### Extract´s composition identification

Based on the previous results, we were interested in identifying which compounds were present within the extracts and could be related to the observed data. In this way, GC-MS identification at the EpSH extract revealed the existence of six compounds, with 69.83% of the total sample amount previously reported with biological activity (Table S4). The most abundant compounds within this sample correspond to alkanes, representing 40.92% of the full sample. On the other hand, ErSA extract has Lanosterol acetate triterpene as a majoritarian compound (99.9%; Table S5). These triterpenes have been recently reported to possess anti-inflammatory and/ or antitumor activities [45].

## Discussion

Complete extracts have been used as nutritional supplements and nutraceuticals because, generally, the effect is not generated with a single component but with a set of them, which can cause a synergic effect on specific biological targets [46–48]. In this context, Cho et al. (2011) previously mentioned that using sub-fractionated extracts with different solvents improves their bioactive properties by concentrating the phytoconstituents and, thus, enhancing their effects [46]. In this way, once the solvent is evaporated, the obtained extract concentrates different phytocomponents, i.e., the hexane extraction solubilized lipophilic compounds as lipids and essential oils; the dichloromethane extraction solubilized terpenoids and sterols; the ethyl acetate extraction solubilized principally low molecular weight flavonoids as phenolic acids, flavonols, and anthocyanins; and finally, the ethanol extraction solubilized fundamentally polyphenols [49–51].

Concerning those phytoconstituents presenting antioxidant capacity, there are not many studies in the Escallonia genus, but compared to the work carried out by Simirgiotis et al. (2012) [52], in our research, we got a higher concentration of phytoconstituents and an improved total antioxidant capacity. This may be due to the extraction method, the solvents, and/or the different organs used to obtain the extracts. Particularly in the extraction method, the SLE-UAE methodology (Solid liquid extraction-ultrasound-assisted extraction) used in this research has several advantageous properties: (1) selectivity in the extraction of bioactive compounds; (2) increased concentration of phytoconstituents with antioxidant capacity as polyphenols; (3) short time of extraction; (4) amelioration of extraction efficiency [51, 53–55]. Further, the plant´s separation by parts (organs) allows the obtention of different secondary metabolites [24].

**Fig. 3:**
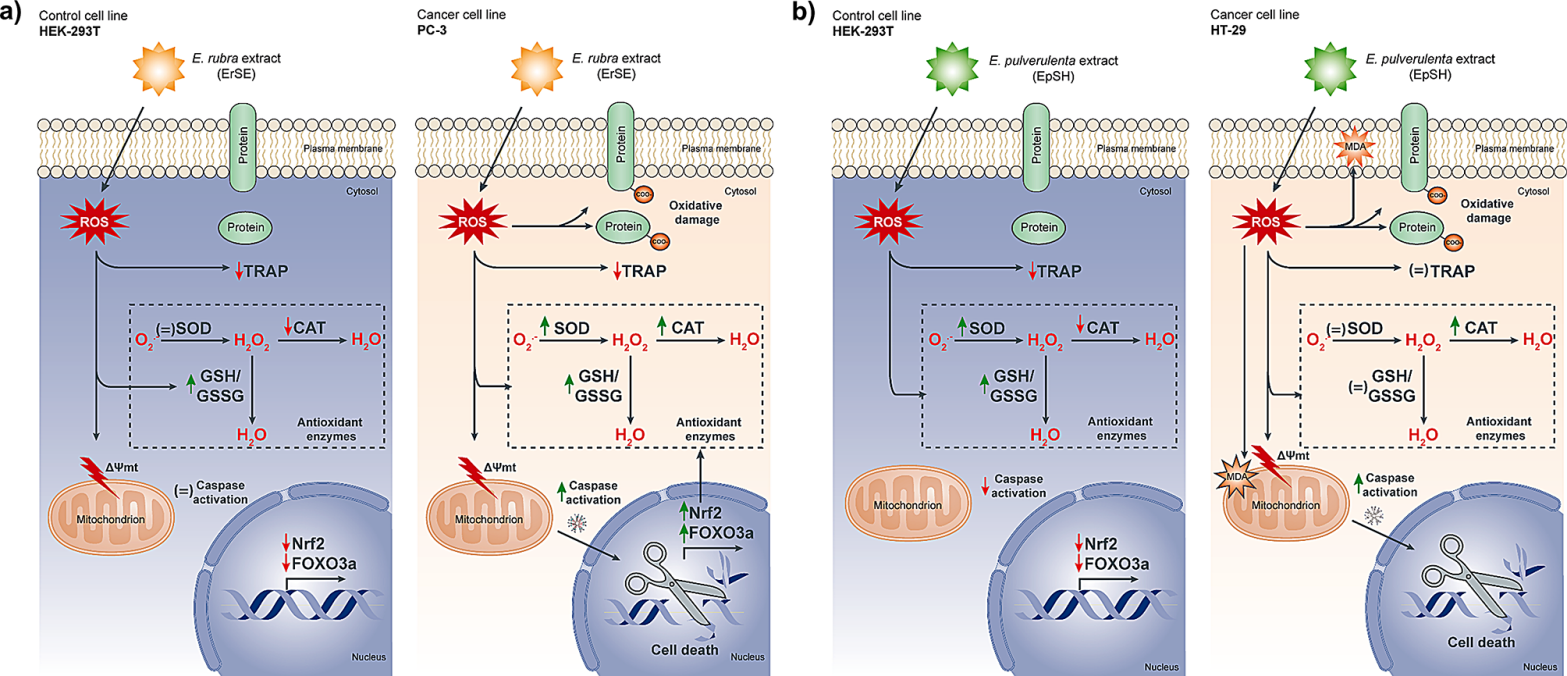
Schematic summary of the main findings of this work. (**a**) ErSA selectivity for the prostate cancer (PC-3) cell line; and (**b**) EpSH extract selectivity for the colon cancer (HT-29) cell line. In both cases, when compared to the non-tumor cell line (HEK-293T). Symbols represent the obtained results compared to the control of each experiment; (=): no change compared with the control; Green arrow: increase compared with the control; Red arrow: decrease compared with the control; ROS: reactive oxygen species; TRAP: total reactive antioxidant power; MDA: lipoperoxidation (malondialdehyde); SOD: superoxide dismutase; CAT: catalase; GSH/GSSG: GSH/GSSG ratio; O_2_.-: superoxide radical; H_2_O_2_: hydrogen peroxide; H_2_O: wáter; ΔΨmt: mitochondrial membrane potential; Nrf2: nuclear factor erythroid 2–related factor 2; FOXO3a: transcription factor forkhead box O-3 a

Among the 36 extracts analyzed, 20 had an EC_50_ ≤ 10 µg*mL^− 1^. Manosroi et al. (2006) suggest that EC_50_ values less than 125 µg*mL^− 1^ might be potential candidates for developing anticancer therapeutic agents [56]. Among these 20 extracts, only 2 were non-cytotoxic to our control nontumoral cancer cell line (HEK-293T). Further, selectivity is the essential feature of an effective anticancer drug [57], which can be obtained through the selectivity index (SI) and by comparing it against gold standard drugs (as in this work). Previous research has pointed out that a SI > 2 is a promissory value [58]. Then, considering the obtained extracts, both are good candidates for developing new drug therapies against cancer, where ErSA and EpSH show selectivity against PC-3 (prostate) and HT-29 (colon) cancer cell lines, respectively. Additionally, based on the obtained results, it is possible to establish both extracts’ oxidative stress action mechanism (selective oxycution), as shown in Fig. 3. As previously stated, ROS are implicated in various stages of cancer development [5, 12, 59–61]. In this respect, cancer cells might be more sensitive to changes in redox homeostasis than normal cells due to changes in the production of ROS or the levels of antioxidant defenses, thus becoming a potential therapeutic target. Therefore, the ROS increase over basal levels can lead to a variation in the antioxidant defense efficiency and, consequently, selectively kill cancer cell lines without harming the normal cells through a phenomenon called “selective oxycution” [5, 9, 12, 62].

The GC-MS analysis allows us to identify the compounds within the two extracts to deepen our studies. Related to the ErSA extract, it is known that lanosterol modulates the immune response [63], has an antiproliferative effect on daunorubicin-resistant leukemia cell line (CEM/R2) [64], and can activate the antioxidant defenses through the Nrf2 transcription factor [65], as the observed results for the ErSA extract in this work. On the other hand, regarding the EpSH extract, it has been previously described that eicosanes have antitumoral activity in different cancer cell lines [66]. Also, the triacontanes present antibacterial, antidiabetic, and antitumoral activity [67, 68]. Further, apigenin derivatives are related to antioxidant activity, promoting the Nrf2 expression and the increase in antioxidant enzymes (GSH-synthetase, catalase, and SOD) and the inhibition of ROS-producing enzymes as NOX [69–71]. Moreover, apigenin derivatives affect metastasis and angiogenesis in oral cancer [72, 73]. Additionally, ɣ-sitosterol is associated with cycle arrest and proapoptotic effect in breast and lung cancer cell lines [74]. Finally, bisabolol derivatives have been described as antitumoral compounds, promoting ROS production and loss of mitochondrial integrity [75] and decreasing cell proliferation and viability in pancreatic cancer cell lines [76–78]. This is similar to how EpSH extract affects the HT-29 cell line.

## Conclusions

In summary, we have obtained 36 extracts with antioxidant capacity linked to their polar characteristic. Moreover, two extracts stand out with selective cytotoxicity for colon (HT-29) and prostate (PC-3) cancer cell lines. Further, both extracts showed “selective oxycution” effects, generating oxidative stress and activating regulated death pathways in cancer cell lines without affecting the control non-tumor cell line. These results indicate that ErSA and EpSH are potential candidates to develop further research against cancer; thus, future directions should be to employ more complex models, such as 3D cultures and animal models, allowing more evidence to be obtained for developing new therapeutic solutions against this pathology. Finally, our findings shed new light on the chemical and biological understanding of the ancestral native flora, a source of uncountable and unknown bioactive compounds.

### Electronic supplementary material

Below is the link to the electronic supplementary material.

**Supplementary Material 1: Supplementary Table S1.** Phytoconstituents concentration per extract. **Supplementary Table S2.** Total antioxidant capacity per extract. **Supplementary Table S3.** Selectivity Index results. **Supplementary Figure S1.** Selected cells’ enzyme activities when exposed to selected extracts. **Supplementary Figure S2.** Reduced glutathione/oxidized glutathione ratio and cell total antioxidant capacity for selected cell lines when exposed to two extracts. **Supplementary Figure S3.** Oxidative damage measured through lipoperoxidation and carbonyl concentration for selected cell lines when exposed to two extracts. **Supplementary Figure S4.** Comparative histogram representing the number of events versus DCF, Rho123, and FITC-VAD-FMK fluorescence for selected cell lines when exposed to two extracts at different concentrations. **Supplementary Table S4.** GC-MS identification of EpSH. **Supplementary Table S5.** GC-MS identification of ErSE

## Data Availability

The datasets used and/or analyzed during the current study are available from the corresponding author on reasonable request.

## References

[1] Mailloux RJ (2015). Teaching the fundamentals of electron transfer reactions in mitochondria and the production and detection of reactive oxygen species. Redox Biol.

[2] Valko M, Rhodes CJ, Moncol J, Izakovic M, Mazur M (2006). Free radicals, metals and antioxidants in oxidative stress-induced cancer. Chemico-Biol Interact.

[3] Kalyanaraman B (2013). Teaching the basics of redox biology to medical and graduate students: oxidants, antioxidants and disease mechanisms. Redox Biol.

[4] Sabharwal SS, Schumacker PT (2014). Mitochondrial ROS in cancer: initiators, amplifiers or an Achilles’ heel?. Nat Rev Cancer.

[5] Sosa V, Moliné T, Somoza R, Paciucci R, Kondoh H, Lleonart ME (2013). Oxidative stress and cancer: an overview. Ageing Res Rev.

[6] Luo J, Solimini NL, Elledge SJ (2009). Principles of Cancer Therapy: Oncogene and Non-oncogene Addiction. Cell.

[7] Liou G-Y, Storz P (2010). Reactive oxygen species in cancer. Free Radic Res.

[8] Reuter S, Gupta SC, Chaturvedi MM, Aggarwal BB (2010). Oxidative stress, inflammation, and cancer: how are they linked?. Free Radic Biol Med.

[9] Burgess DJ, Selective (2011). Oxycution? Nat Reviews Cancer.

[10] Pelicano H, Carney D, Huang P (2004). ROS stress in cancer cells and therapeutic implications. Drug Resist Updates.

[11] Trachootham D, Alexandre J, Huang P (2009). Targeting cancer cells by ROS-mediated mechanisms: a radical therapeutic approach?. Nat Rev Drug Discovery.

[12] Gorrini C, Harris IS, Mak TW (2013). Modulation of oxidative stress as an anticancer strategy. Nat Rev Drug Discovery.

[13] Poillet-Perez L, Despouy G, Delage-Mourroux R, Boyer-Guittaut M (2015). Interplay between ROS and autophagy in cancer cells, from tumor initiation to cancer therapy. Redox Biol.

[14] Trachootham D, Zhou Y, Zhang H, Demizu Y, Chen Z, Pelicano H, et al. Selective killing of oncogenically transformed cells through a ROS-mediated mechanism by beta-phenylethyl isothiocyanate. Cancer Cell. 2006;10(3):241–52. 10.1016/j.ccr.2006.08.009 16959615

[15] Juan ME, Wenzel U, Daniel H, Planas JM (2008). Resveratrol induces apoptosis through ROS-Dependent Mitochondria pathway in HT-29 human colorectal carcinoma cells. J Agric Food Chem.

[16] Villagrán C (1998). Etnobotánica indígena De Los bosques de Chile: sistema de clasificación de un recurso de uso múltiple. Revista Chil de Historia Nat.

[17] Garcia R, Erazo S, Canepa A, Lemus I, Erazo S (1990). Secondary metabolites of Escallonia Illinita Presl. An Real Acad Farm.

[18] Hoffman P. Herbolaria Y nutrición natural: la Salud Al Alcance De Todos. Editorial Pax México; 2005.

[19] García NOC (2008). Árboles Nativos De Chile.

[20] Hoffmann A (2012). Flora Silvestre De Chile, zona central.

[21] Jara C, Leyton M, Osorio M, Silva V, Fleming F, Paz M (2017). Antioxidant, phenolic and antifungal profiles of Acanthus mollis (Acanthaceae). Nat Prod Res.

[22] Waterman P, Mole S. Methods in ecology: analysis of phenolic plant metabolites. Victoria/Australia: Blackwell Scientific Publicatios. 1994:74–93.

[23] Arvouet-Grand A, Vennat B, Pourrat A, Legret P (1994). [Standardization of propolis extract and identification of principal constituents]. J Pharm Belg.

[24] Mellado M, Madrid A, Jara C, Espinoza L (2012). Antioxidant effects of muehlenbeckia hastulata j. (polygonaceae) extracts. J Chil Chem Soc.

[25] Romay C, Pascual C, Lissi EA (1996). The reaction between ABTS radical cation and antioxidants and its use to evaluate the antioxidant status of serum samples. Braz J Med Biol Res.

[26] Brand-Williams W, Cuvelier ME, Berset C (1995). Use of a free radical method to evaluate antioxidant activity. LWT - Food Science and Technology.

[27] Ruch RJ, Cheng SJ, Klaunig JE (1989). Prevention of cytotoxicity and inhibition of intercellular communication by antioxidant catechins isolated from Chinese green tea. Carcinogenesis.

[28] Dudonné S, Vitrac X, Coutière P, Woillez M, Mérillon J-M (2009). Comparative Study of Antioxidant Properties and total phenolic content of 30 plant extracts of Industrial Interest using DPPH, ABTS, FRAP, SOD, and ORAC assays. J Agric Food Chem.

[29] Montenegro I, Villegas AM, Zaror L, Martinez R, Werner E, Carrasco-Altamirano H (2012). Antimicrobial activity of ethyl acetate extract and essential oil from bark of Laurelia sempervirens against multiresistant bacteria. Boletín Latinoamericano Y Del Caribe De Plantas Medicinales Y Aromáticas.

[30] Canales N, Montenegro I, Párraga M, Olguín Y, Godoy P, Werner E (2016). In Vitro Antimicrobial activity of Embothrium coccineum used as Traditional Medicine in Patagonia against multiresistant Bacteria. Molecules.

[31] NIST NIoSaT. NIST/EPA/NIH Mass Spectral Library with Search Program (Data Version: NIST 11, Software Version 2.0 g) 2022. Available from: http://webbook.nist.gov/chemistry/name-ser.html.

[32] Santander R, Creixell W, Sánchez E, Tomic G, Silva JR, Acevedo CA (2013). Recognizing age at slaughter of cattle from beef samples using GC/MS–SPME chromatographic method. Food Bioprocess Technol.

[33] Skehan P, Storeng R, Scudiero D, Monks A, McMahon J, Vistica D (1990). New colorimetric cytotoxicity assay for anticancer-drug screening. J Natl Cancer Inst.

[34] Koch A, Tamez P, Pezzuto J, Soejarto D (2005). Evaluation of plants used for antimalarial treatment by the Maasai of Kenya. J Ethnopharmacol.

[35] Lowry O, Rosebrough N, Farr AL, Randall R (1951). PROTEIN MEASUREMENT WITH THE FOLIN PHENOL REAGENT. J Biol Chem.

[36] Beauchamp C, Fridovich I (1971). Superoxide dismutase: improved assays and an assay applicable to acrylamide gels. Anal Biochem.

[37] Aebi H. [13] catalase in vitro. Methods in enzymology. Volume 105. Academic Press; 1984. pp. 121–6. 10.1016/s0076-6879(84)05016-3 6727660

[38] Rahman I, Kode A, Biswas SK (2006). Assay for quantitative determination of glutathione and glutathione disulfide levels using enzymatic recycling method. Nat Protoc.

[39] Esterbauer H, Cheeseman KH, Dianzani MU, Poli G, Slater TF (1982). Separation and characterization of the aldehydic products of lipid peroxidation stimulated by ADP-Fe2 + in rat liver microsomes. Biochem J.

[40] Levine RL, Garland D, Oliver CN, Amici A, Climent I, Lenz A-G, et al. [49] determination of carbonyl content in oxidatively modified proteins. Methods in enzymology. Volume 186. Academic Press; 1990. pp. 464–78. 10.1016/0076-6879(90)86141-h 1978225

[41] Rothe G, Valet G (1990). Flow Cytometric Analysis of Respiratory Burst Activity in Phagocytes with Hydroethidine and 2′,7′-Dichlorofluorescin. J Leukoc Biol.

[42] Ferlini C, Scambia G (2007). Assay for apoptosis using the mitochondrial probes, Rhodamine123 and 10-N-nonyl acridine orange. Nat Protoc.

[43] Yang T, Witham TF, Villa L, Erff M, Attanucci J, Watkins S (2002). Glioma-associated hyaluronan induces apoptosis in dendritic cells via inducible nitric oxide synthase: implications for the use of dendritic cells for therapy of gliomas. Cancer Res.

[44] Zamzami N, Larochette N, Kroemer G (2005). Mitochondrial permeability transition in apoptosis and necrosis. Cell Death & Differentiation.

[45] Lima E, Medeiros J (2020). Terpenoid compounds in the latex of Euphorbia Azorica from Azores. Biomed J Sci & Tech Res.

[46] Cho M, Lee H-S, Kang I-J, Won M-H, You S (2011). Antioxidant properties of extract and fractions from Enteromorpha prolifera, a type of green seaweed. Food Chem.

[47] Du W-X, Olsen CW, Avena-Bustillos RJ, Friedman M, McHugh TH (2011). Physical and Antibacterial Properties of Edible Films Formulated with Apple skin polyphenols. J Food Sci.

[48] Elfalleh W, Tlili N, Nasri N, Yahia Y, Hannachi H, Chaira N (2011). Antioxidant capacities of Phenolic compounds and tocopherols from Tunisian pomegranate (Punica granatum) Fruits. J Food Sci.

[49] Seidel V, Sarker SD, Nahar L (2012). Initial and bulk extraction of Natural products isolation. Natural products isolation.

[50] Azmir J, Zaidul ISM, Rahman MM, Sharif KM, Mohamed A, Sahena F (2013). Techniques for extraction of bioactive compounds from plant materials: a review. J Food Eng.

[51] Koçak E, Pazır F (2018). Effect of extraction methods on Bioactive compounds of Plant Origin. Turkish J Agriculture-Food Sci Technol.

[52] Simirgiotis MJ, Silva M, Becerra J, Schmeda-Hirschmann G (2012). Direct characterisation of phenolic antioxidants in infusions from four Mapuche medicinal plants by liquid chromatography with diode array detection (HPLC-DAD) and electrospray ionisation tandem mass spectrometry (HPLC-ESI–MS). Food Chem.

[53] Zhang Q-W, Lin L-G, Ye W-C (2018). Techniques for extraction and isolation of natural products: a comprehensive review. Chin Med.

[54] Barba FJ, Zhu Z, Koubaa M, Sant’Ana AS, Orlien V (2016). Green alternative methods for the extraction of antioxidant bioactive compounds from winery wastes and by-products: a review. Trends Food Sci Technol.

[55] Chemat F, Rombaut N, Sicaire A-G, Meullemiestre A, Fabiano-Tixier A-S, Abert-Vian M (2017). Ultrasound assisted extraction of food and natural products. Mechanisms, techniques, combinations, protocols and applications. A review. Ultrason Sonochem.

[56] Manosroi J, Dhumtanom P, Manosroi A (2006). Anti-proliferative activity of essential oil extracted from Thai medicinal plants on KB and P388 cell lines. Cancer Lett.

[57] Lopez-Lazaro M. Experimental cancer pharmacology for researchers: at what concentration should my drug kill cancer cells so that it has potential for cancer therapy? Amazon Digital Services. Inc ASIN: B00MMO25NM ed. 2014.

[58] Badisa RB, Darling-Reed SF, Joseph P, Cooperwood JS, Latinwo LM, Goodman CB (2009). Selective cytotoxic activities of two novel synthetic drugs on human breast carcinoma MCF-7 cells. Anticancer Res.

[59] Liu J, Wang Z (2015). Increased oxidative stress as a selective anticancer therapy. Oxidative Med Cell Longev.

[60] Kim J, Kim J, Bae J-S (2016). ROS homeostasis and metabolism: a critical liaison for cancer therapy. Exp Mol Med.

[61] Panieri E, Santoro MM (2016). ROS homeostasis and metabolism: a dangerous liason in cancer cells. Cell Death Dis.

[62] Diehn M, Cho RW, Lobo NA, Kalisky T, Dorie MJ, Kulp AN (2009). Association of reactive oxygen species levels and radioresistance in cancer stem cells. Nature.

[63] Araldi E, Fernández-Fuertes M, Canfrán-Duque A, Tang W, Cline GW, Madrigal-Matute J (2017). Lanosterol modulates TLR4-Mediated Innate Immune responses in macrophages. Cell Rep.

[64] Stäubert C, Krakowsky R, Bhuiyan H, Witek B, Lindahl A, Broom O (2015). Increased lanosterol turnover: a metabolic burden for daunorubicin-resistant leukemia cells. Med Oncol.

[65] Gill BS, Kumar S, Navgeet (2017). Ganoderic acid targeting nuclear factor erythroid 2–related factor 2 in lung cancer. Tumor Biology.

[66] Walia M, Mann TS, Kumar D, Agnihotri VK, Singh B (2012). Chemical composition and in Vitro cytotoxic activity of essential oil of leaves of Malus domestica growing in Western Himalaya (India). Evid Based Complement Alternat Med.

[67] Xu J, Han Q-B, Li S-L, Chen X-J, Wang X-N, Zhao Z-Z (2013). Chemistry, bioactivity and quality control of Dendrobium, a commonly used tonic herb in traditional Chinese medicine. Phytochem Rev.

[68] Paudel MR, Chand MB, Pant B, Pant B (2019). Assessment of antioxidant and cytotoxic activities of extracts of Dendrobium Crepidatum. Biomolecules.

[69] Huang C-S, Lii C-K, Lin A-H, Yeh Y-W, Yao H-T, Li C-C (2013). Protection by chrysin, apigenin, and luteolin against oxidative stress is mediated by the Nrf2-dependent up-regulation of heme oxygenase 1 and glutamate cysteine ligase in rat primary hepatocytes. Arch Toxicol.

[70] Paredes-Gonzalez X, Fuentes F, Jeffery S, Saw CL-L, Shu L, Su Z-Y (2015). Induction of NRF2-mediated gene expression by dietary phytochemical flavones apigenin and luteolin. Biopharm Drug Dispos.

[71] Telange DR, Patil AT, Pethe AM, Fegade H, Anand S, Dave VS (2017). Formulation and characterization of an apigenin-phospholipid phytosome (APLC) for improved solubility, in vivo bioavailability, and antioxidant potential. Eur J Pharm Sci.

[72] Peng Q, Deng Z, Pan H, Gu L, Liu O, Tang Z (2018). Mitogen-activated protein kinase signaling pathway in oral cancer (review). Oncol Lett.

[73] Salehi B, Venditti A, Sharifi-Rad M, Kręgiel D, Sharifi-Rad J, Durazzo A (2019). The Therapeutic Potential of Apigenin International Journal of Molecular Sciences.

[74] Sundarraj S, Thangam R, Sreevani V, Kaveri K, Gunasekaran P, Achiraman S (2012). γ-Sitosterol from Acacia nilotica L. induces G2/M cell cycle arrest and apoptosis through c-Myc suppression in MCF-7 and A549 cells. J Ethnopharmacol.

[75] Cavalieri E, Bergamini C, Mariotto S, Leoni S, Perbellini L, Darra E (2009). Involvement of mitochondrial permeability transition pore opening in α-bisabolol induced apoptosis. FEBS J.

[76] Seki T, Kokuryo T, Yokoyama Y, Suzuki H, Itatsu K, Nakagawa A (2011). Antitumor effects of α-bisabolol against pancreatic cancer. Cancer Sci.

[77] Uno M, Kokuryo T, Yokoyama Y, Senga T, Nagino M (2016). α-Bisabolol inhibits invasiveness and motility in pancreatic Cancer through KISS1R activation. Anticancer Res.

[78] Murata Y, Kokuryo T, Yokoyama Y, Yamaguchi J, Miwa T, Shibuya M (2017). The Anticancer effects of Novel α-Bisabolol derivatives against pancreatic Cancer. Anticancer Res.

