# Supplementary material for: Oxidative stress promotes cytotoxicity in human cancer cell lines exposed to Escallonia spp. extracts
Source: BMC Complement Med Ther. 2024 Jan 13;24:38. doi: 10.1186/s12906-024-04341-4 (PMC10787448; doi:10.1186/s12906-024-04341-4)
Supplement: Supplementary file 1 — Supplementary Material 1: Supplementary Table S1. Phytoconstituents concentration per extract. Supplementary Table S2. Total antioxidant capacity per extract. Supplementary Table S3. Selectivity Index results. Supplementary Figure S1. Selected cells’ enzyme activities when exposed to selected extracts. Supplementary Figure S2. Reduced glutathione/oxidized glutathione ratio and cell total antioxidant capacity for selected cell lines when exposed to two extracts. Supplementary Figure S3. Oxidative damage measured through lipoperoxidation and carbonyl concentration for selected cell lines when exposed to two extracts. Supplementary Figure S4. Comparative histogram representing the number of events versus DCF, Rho123, and FITC-VAD-FMK fluorescence for selected cell lines when exposed to two extracts at different concentrations. Supplementary Table S4. GC-MS identification of EpSH. Supplementary Table S5. GC-MS identification of ErSE [file 12906_2024_4341_MOESM1_ESM.docx]

# *Supplementary Information*

**Oxidative stress promotes cytotoxicity in human cancer cell lines exposed to *Escallonia spp.* Extracts**

Carlos Jara-Gutiérrez^1,2^, Luis Mercado^3^, Marilyn Paz-Araos^1,2^, Carolyn Howard^2^, Mario Parraga^1^, Camila Escobar^1^, Marco Mellado^4^, Alejandro Madrid^5^, Iván Montenegro^1^, Paula Santana^6^, Paola Murgas^7^, Cristina Jimenez-Jara^8^, Luis Guillermo González-Olivares^9^, Manuel Ahumada^10,11,^*, Joan Villena^1,^*

^1^Centro de Investigaciones Biomédicas (CIB), Facultad de Medicina, Universidad de Valparaíso, Valparaíso, Chile.

^2^Facultad de Medicina, Escuela de Kinesiología, Universidad de Valparaíso, Valparaíso, Chile.

^3^Laboratorio de Genética e Inmunología Molecular, Instituto de Biología, Pontificia Universidad Católica de Valparaíso, Chile.

^4^Instituto de Investigación y Postgrado, Facultad de Ciencias de la Salud, Universidad Central de Chile, 8330507 Santiago, Chile.

^5^Laboratorio de Productos Naturales y Síntesis Orgánica (LPNSO), Departamento de Ciencias y Geografía, Facultad de Ciencias Naturales y Exactas, Universidad de Playa Ancha, Avda. Leopoldo Carvallo 270, Playa Ancha, Valparaíso 2340000, Chile.

^6^Instituto de Ciencias Químicas Aplicadas, Facultad de Ingeniería, Universidad Autónoma de Chile, el Llano Subercaseaux 2801, San Miguel, Santiago, Chile.

^7^Instituto de Bioquímica y Microbiología, Facultad de Ciencias, Center for Interdisciplinary Studies on the Nervous System (CISNe), Universidad Austral de Chile, Valdivia, Chile.

^8^Doctorado en Ciencias e Ingeniería para la Salud, Universidad de Valparaíso, Chile.

^9^Academic Area of Chemistry, Universidad Autónoma del Estado de Hidalgo, Hidalgo, Mineral de la Reforma, Mexico.

^10^Centro de Nanotecnología Aplicada, Facultad de Ciencias, Ingeniería y Tecnología, Universidad Mayor, Santiago, Chile.

^11^Escuela de Biotecnología, Facultad de Ciencias, Ingeniería y Tecnología, Universidad Mayor, Santiago, Chile.

*[juan.villena@uv.cl](mailto:juan.villena@uv.cl)

^*^[manuel.ahumada@umayor.cl](mailto:manuel.ahumada@umayor.cl)

| **Content** |  | **Page** |
| --- | --- | --- |
| This page | …………………………………………………..………………………………………………….. | S1 |
| Table S1 | …………………………………………………..………………………………………………….. | S2 |
| Table S2 | …………………………………………………..………………………………………………….. | S3 |
| Table S3 | …………………………………………………..………………………………………………….. | S4 |
| Figure S1 | …………………………………………………..………………………………………………….. | S5 |
| Figure S2 | …………………………………………………..………………………………………………….. | S6 |
| Figure S3 | …………………………………………………..………………………………………………….. | S7 |
| Figure S4 | …………………………………………………..………………………………………………….. | S8 |
| Table S4 | …………………………………………………..………………………………………………….. | S9 |
| Table S5 | …………………………………………………..………………………………………………….. | S9 |

**Table S1.** Phytoconstituents concentration per extract (total phenols content, total flavonoids content, and total anthraquinones content). The results showed mean values ± standard deviation (n=3).

| **Plant** | **Organ** | **Extract** | **Total phenols (GAE mM)** | **Total Flavonoids (QE mM)** | **Total anthraquinones (EE mM)** |
| --- | --- | --- | --- | --- | --- |
| *E. illinita* | Stem | H | 0.1319 ± 0.0036 | 0.0460 ± 0.0003 | 0.030 ± 0.0008^a^ |
|  |  | D | 0.3701 ± 0.0053^a^ | 0.1844 ± 0.0072^a^ | 0.022 ± 0.0003^b^ |
|  |  | A | 0.4253 ± 0.0043^a^ | 0.1736 ± 0.0080^a^ | 0.026 ± 0.0008^b^ |
|  |  | E | 0.2368 ± 0.0225^b^ | 0.0360 ± 0.0010 | 0.015 ± 0.0005 |
|  | Leaves | H | 0.2082 ± 0.0107^b^ | 0.2483 ± 0.0046^b^ | 0.049 ± 0.0002^c^ |
|  |  | D | 0.2268 ± 0.0104^b^ | 0.6189 ± 0.0352^c^ | 0.036 ± 0.0005^a^ |
|  |  | A | 0.4202 ± 0.0058^a^ | 0.7032 ± 0.0001^c^ | 0.047 ± 0.0008^c^ |
|  |  | E | 0.2616 ± 0.0054^b^ | 0.0632 ± 0.0018 | 0.016 ± 0.0005 |
| *E. rubra* | Stem | H | 0.0984 ± 0.0111 | 0.0374 ± 0.0018 | 0.039 ± 0.0004^a^ |
|  |  | D | 0.1238 ± 0.0037 | 0.0173 ± 0.0009 | 0.017 ± 0.0010 |
|  |  | A | 0.1613 ± 0.0003 | 0.0406 ± 0.0014 | 0.026 ± 0.0008^b^ |
|  |  | E | 0.1683 ± 0.0175 | 0.0372 ± 0.0002 | 0.028 ± 0.0004^b^ |
|  | Leaves | H | 0.1492 ± 0.0368 | 0.0552 ± 0.0003 | 0.048 ± 0.0007^c^ |
|  |  | D | 0.1823 ± 0.0115^b^ | 0.1800 ± 0.0002^a^ | 0.066 ± 0.0004^d^ |
|  |  | A | 0.3499 ± 0.0423^a^ | 0.2512 ± 0.0013^b^ | 0.049 ± 0.0001^c^ |
|  |  | E | 0.3146 ± 0.0085^a^ | 0.1551 ± 0.0070^a^ | 0.032 ± 0.0002^a^ |
| *E. revoluta* | Stem | H | 0.0899 ± 0.0246 | 0.0945 ± 0.0029 | 0.077 ± 0.0002^e^ |
|  |  | D | 0.1260 ± 0.0034 | 0.0324 ± 0.0001 | 0.029 ± 0.0001^b^ |
|  |  | A | 0.2030 ± 0.0010^b^ | 0.0458 ± 0.0004 | 0.027 ± 0.0001^b^ |
|  |  | E | 0.3492 ± 0.0143^a^ | 0.0465 ± 0.0016 | 0.025 ± 0.0006^b^ |
|  | Leaves | H | 0.0917 ± 0.0006 | 0.0725 ± 0.0001 | 0.060 ± 0.0006^d^ |
|  |  | D | 0.2161 ± 0.0088^b^ | 0.1453 ± 0.0014^a^ | 0.065 ± 0.0007^d^ |
|  |  | A | 0.4206 ± 0.0062^a^ | 0.2622 ± 0.0016^b^ | 0.040 ± 0.0003^c^ |
|  |  | E | 0.2753 ± 0.0029^b^ | 0.1144 ± 0.0011^a^ | 0.029 ± 0.0002^b^ |
| *E. pulverulenta* | Stem | H | 0.2190 ± 0.0167^b^ | 0.0472 ± 0.0005 | 0.018 ± 0.0001 |
|  |  | D | 0.2208 ± 0.0124^b^ | 0.1357 ± 0.0014^a^ | 0.024 ± 0.0002^b^ |
|  |  | A | 0.2666 ± 0.0217^b^ | 0.1562 ± 0.0060^a^ | 0.030 ± 0.0002^a^ |
|  |  | E | 0.3605 ± 0.0009^a^ | 0.1214 ± 0.0012^a^ | 0.029 ± 0.0007^b^ |
|  | Leaves | H | 0.1575 ± 0.0059 | 0.0270 ± 0.0022 | 0.023 ± 0.0011^b^ |
|  |  | D | 0.2963 ± 0.0038^b^ | 0.0595 ± 0.0001 | 0.044 ± 0.0002^c^ |
|  |  | A | 0.4778 ± 0.0046^a^ | 0.2529 ± 0.0022^b^ | 0.037 ± 0.0017^a^ |
|  |  | E | 0.7015 ± 0.0203^c^ | 0.2332 ± 0.0006^b^ | 0.032 ± 0.0003^a^ |
|  | Flowers | H | 0.1492 ± 0.0173 | 0.0272 ± 0.0021 | 0.014 ± 0.0008 |
|  |  | D | 0.3571 ± 0.0082^a^ | 0.1929 ± 0.0019^a^ | 0.017 ± 0.0004 |
|  |  | A | 0.4164 ± 0.0106^a^ | 0.3139 ± 0.0153^d^ | 0.019 ± 0.0006 |
|  |  | E | 0.6461 ± 0.0057^c^ | 0.2961 ± 0.0116^d^ | 0.022 ± 0.0009^b^ |

Super index letters show significant differences p˂0.05.

**Table S2.** Total antioxidant capacity per extracts (TRAP= ABTS• radical scavenger, DPPH= DPPH• radical scavenger, H_2_O_2_= H_2_O_2_ scavenger, FRAP= ferric ion reductor capacity). The results showed mean values ± standard deviation (n=3) to the different species of Escallonia genus (*E. illinita*, *E. rubra*, *E. revoluta* y *E. pulverulenta*), to different organs (stem, leaves and flowers) and the different extractions (H= n-hexane, D= dichloromethane, A= ethyl acetate and E= ethanol).

| **Plant** | **Organ** | **Extract** | **ABTS^.^ scavenging activity**  **(TEAC mM)** | **DPPH^.^ scavenging activity**  **(IC_50_ mg*mL^-1^)** | **H_2_O_2_ scavenging activity**  **(IC_50_ mg*mL^-1^)** | **Ferric Reducing Antioxidant**  **Capacity**  **(TEAC mM)** |
| --- | --- | --- | --- | --- | --- | --- |
| *E. illinita* | Stem | H | 0.0029 ± 0.0005^a^ | 9.8628 ± 0.1774^a^ | 2.0757 ± 0.0208^a^ | 0.0002 ± 0.00003^a^ |
|  |  | D | 0.0215 ± 0.0032^b^ | 6.3930 ± 0.3611^b^ | 1.3022 ± 0.1473^a^ | 0.0003 ± 0.00001^a^ |
|  |  | A | 0.0234 ± 0.0009^b^ | 2.2189 ± 0.0787^c^ | 2.7326 ± 0.8252^a^ | 0.0004 ± 0.00001^b^ |
|  |  | E | 0.0275 ± 0.0023^b^ | 1.3529 ± 0.0281^d^ | 2.8460 ± 0.2217^a^ | 0.0004 ± 0.00001^b^ |
|  | Leaves | H | 0.0044 ± 0.0001^a^ | 5.5778 ± 0.4235^b^ | 1.6448 ± 0.0511^a^ | 0.0002 ± 0.00004^a^ |
|  |  | D | 0.0102 ± 0.0011^c^ | 41.7800 ± 1.0410^e^ | 1.3683 ± 0.0064^a^ | 0.0002 ± 0.00001^a^ |
|  |  | A | 0.0231 ± 0.0011^b^ | 7.5648 ± 1.0983^b^ | 4.8915 ± 0.6776^b^ | 0.0003 ± 0.00001^a^ |
|  |  | E | 0.0251 ± 0.0007^b^ | 1.4269 ± 0.0080^d^ | 2.8690 ± 0.6854^a^ | 0.0003 ± 0.00001^a^ |
| *E. rubra* | Stem | H | 0.0001 ± 0.0001^a^ | 20.2835 ± 3.9668^f^ | 3.2927 ± 0.2569^a^ | 0.0002 ± 0.00003^a^ |
|  |  | D | 0.0024 ± 0.0001^a^ | 6.1334 ± 0.6619^b^ | 3.5105 ± 0.4836^a^ | 0.0003 ± 0.00001^a^ |
|  |  | A | 0.0049 ± 0.0001^a^ | 2.8588 ± 0.1020^c^ | 2.3099 ± 0.1902^a^ | 0.0002 ± 0.00001^a^ |
|  |  | E | 0.0098 ± 0.0004^c^ | 1.8469 ± 0.0358^d^ | 3.4605 ± 0.4482^a^ | 0.0003 ± 0.00006^a^ |
|  | Leaves | H | 0.0008 ± 0.0002^a^ | 5.6666 ± 0.7169^b^ | 5.0190 ± 0.4856^b^ | 0.0002 ± 0.00001^a^ |
|  |  | D | 0.0038 ± 0.0002^a^ | 5.1080 ± 1.4938^b^ | 8.5334 ± 0.7118^c^ | 0.0003 ± 0.00001^b^ |
|  |  | A | 0.0117 ± 0.0001^c^ | 2.0606 ± 0.0549^c^ | 3.2501 ± 0.0860^a^ | 0.0002 ± 0.00007^a^ |
|  |  | E | 0.0238 ± 0.0012^b^ | 1.5428 ± 0.0202^d^ | 3.4846 ± 0.8051^a^ | 0.0003 ± 0.00006^b^ |
| *E. revoluta* | Stem | H | 0.0001 ± 0.0001^a^ | 6.5000 ± 0.6845^b^ | 2.9917 ± 0.0826^a^ | 0.0002 ± 0.00003^c^ |
|  |  | D | 0.0024 ± 0.0003^a^ | 26.2418 ± 5.2140^f^ | 4.0433 ± 0.5865^a^ | 0.0003 ± 0.00003^a^ |
|  |  | A | 0.0099 ± 0.0008^c^ | 2.3149 ± 0.0493^c^ | 5.0766 ± 0.7110^b^ | 0.0003 ± 0.00004^a^ |
|  |  | E | 0.0408 ± 0.0004^d^ | 1.3418 ± 0.0143^d^ | 14.0335 ± 0.3767^d^ | 0.0003 ± 0.00009^a^ |
|  | Leaves | H | 0.0013 ± 0.0001^a^ | 5.1733 ± 0.1372^b^ | 2.5793 ± 0.4019^a^ | 0.0002 ± 0.00001^a^ |
|  |  | D | 0.0056 ± 0.0004^a^ | 2.3274 ± 0.1920^c^ | 4.9895 ± 0.9390^b^ | 0.0003 ± 0.00001^a^ |
|  |  | A | 0.0163 ± 0.0018^b^ | 1.8876 ± 0.0220^d^ | 12.7089 ± 0.2739^e^ | 0.0002 ± 0.00001^a^ |
|  |  | E | 0.0192 ± 0.0009^b^ | 1.5041 ± 0.0198^d^ | 3.4163 ± 0.0217^a^ | 0.0003 ± 0.00004^a^ |
| *E. pulverulenta* | Stem | H | 0.0067 ± 0.0007^a^ | 2.6870 ± 0.0086^c^ | 4.3297 ± 0.8085^b^ | 0.0002 ± 0.00001^c^ |
|  |  | D | 0.0116 ± 0.0009^b^ | 1.7618 ± 0.0308^d^ | 7.4811 ± 0.2439^f^ | 0.0003 ± 0.00008^a^ |
|  |  | A | 0.0155 ± 0.0007^b^ | 1.7869 ± 0.0119^d^ | 2.8035 ± 0.0688^a^ | 0.0003 ± 0.00001^a^ |
|  |  | E | 0.0349 ± 0.0015^d^ | 1.4825 ± 0.0078^d^ | 13.9281 ± 0.7090^d^ | 0.0004 ± 0.00001^b^ |
|  | Leaves | H | 0.0026 ± 0.0005^a^ | 4.6184 ± 0.2741^b^ | 4.1001 ± 0.7969^b^ | 0.0002 ± 0.00004^a^ |
|  |  | D | 0.0168 ± 0.0008^b^ | 1.5244 ± 0.0180^d^ | 2.7593 ± 0.4168^a^ | 0.0003 ± 0.00001^a^ |
|  |  | A | 0.0379 ± 0.0049^d^ | 1.3569 ± 0.0040^d^ | 2.7863 ± 0.1092^a^ | 0.0005 ± 0.00002^b^ |
|  |  | E | 0.1089 ± 0.0106^e^ | 1.1974 ± 0.0187^d^ | 4.2554 ± 0.5820^b^ | 0.0008 ± 0.00001^d^ |
|  | Flowers | H | 0.0041 ± 0.0004^a^ | 4.0922 ± 0.1693^b^ | 2.6609 ± 0.8352^a^ | 0.0003 ± 0.00001^a^ |
|  |  | D | 0.0159 ± 0.0004^c^ | 1.3936 ± 0.0112^d^ | 3.3195 ± 0.5077^a^ | 0.0003 ± 0.00001^a^ |
|  |  | A | 0.0225 ± 0.0001^b^ | 1.3145 ± 0.0186^d^ | 2.9785 ± 0.4107^a^ | 0.0004 ± 0.00001^b^ |
|  |  | E | 0.0652 ± 0.0021^f^ | 1.2822 ± 0.0100^d^ | 6.8221 ± 0.6628^f^ | 0.0003 ± 0.00001^a^ |
| Gallic Acid | | | 1.1300 ± 0.0100^g^ | N.A. | N.A. | 1.72000 ± 0.02000^e^ |
| BHT | | | 1.0600 ± 0.0200^h^ | 0.0600 ± 0.0001^g^ | 2.5267 ± 0.0568^g^ | 1.52000 ± 0.07000^f^ |
| Trolox® | | | N.A. | 0.1067 ± 0.0057^g^ | 2.8633 ± 0.0321^g^ | N.A. |

TRAP was expressed in mM Trolox® equivalent antioxidant capacity per g dry weight (TEAC mM), DPPH in mean inhibitory concentration per g dry weight (IC_50_ mg*mL^-1^), H_2_O_2_ in mean inhibitory concentration per g dry weight (IC_50_ mg*mL^-1^) and FRAP expressed in mM Trolox® equivalent antioxidant capacity per g dry weight (TEAC mM). N.A.= not apply. Superindex letters show to significant differences *p*˂0.05.

**Table S3.** Selectivity index (SI) results of the selected extracts of Escallonia on cancer cell lines (MCF-7= breast cancer; HT-29= colon cancer; PC-3= prostate cancer).

| **Plant** | **Organ** | **Extract** | **Selectivity Index (SI)** | | |
| --- | --- | --- | --- | --- | --- |
|  |  |  | **MCF-7** | **HT-29** | **PC-3** |
| *E. illinita* | Stem | D | 0.51 | 0.91 | 0.36 |
|  |  | A | 1.37 | 0.54 | 0.26 |
| *E. rubra* | Stem | H | 1.37 | 1.50 | 1.69 |
|  |  | D | 0.50 | 0.58 | 0.79 |
|  |  | A | 1.15 | 0.56 | 2.19 |
|  | Leaves | H | 0.18 | 0.68 | 1.07 |
|  |  | D | 0.68 | 0.45 | 0.81 |
| *E. revoluta* | Stem | H | 0.35 | 0.58 | 0.18 |
|  |  | D | 0.58 | 0.78 | 0.72 |
|  |  | A | 0.49 | 1.58 | 1.02 |
| *E. pulverulenta* | Stem | H | 0.98 | 2.31 | 1.54 |
|  |  | D | 0.13 | 0.49 | 0.59 |
|  |  | A | 0.49 | 0.49 | 0.51 |
|  | Leaves | H | 1.58 | 1.65 | 1.50 |
|  |  | D | 0.25 | 1.02 | 0.62 |
|  |  | A | 1.19 | 0.79 | 0.79 |
|  | Flowers | H | 0.99 | 0.50 | 0.82 |
|  |  | D | 1.47 | 1.48 | 1.60 |
|  |  | A | 0.57 | 0.84 | 0.58 |
|  |  | E | 0.96 | 1.04 | 0.74 |
| DOXO | | | 5.0 | 10.0 | 2.0 |

SI was obtained from the mean value of EC_50_ of the cell lines. Doxorubicin (DOXO) was used as a positive control.


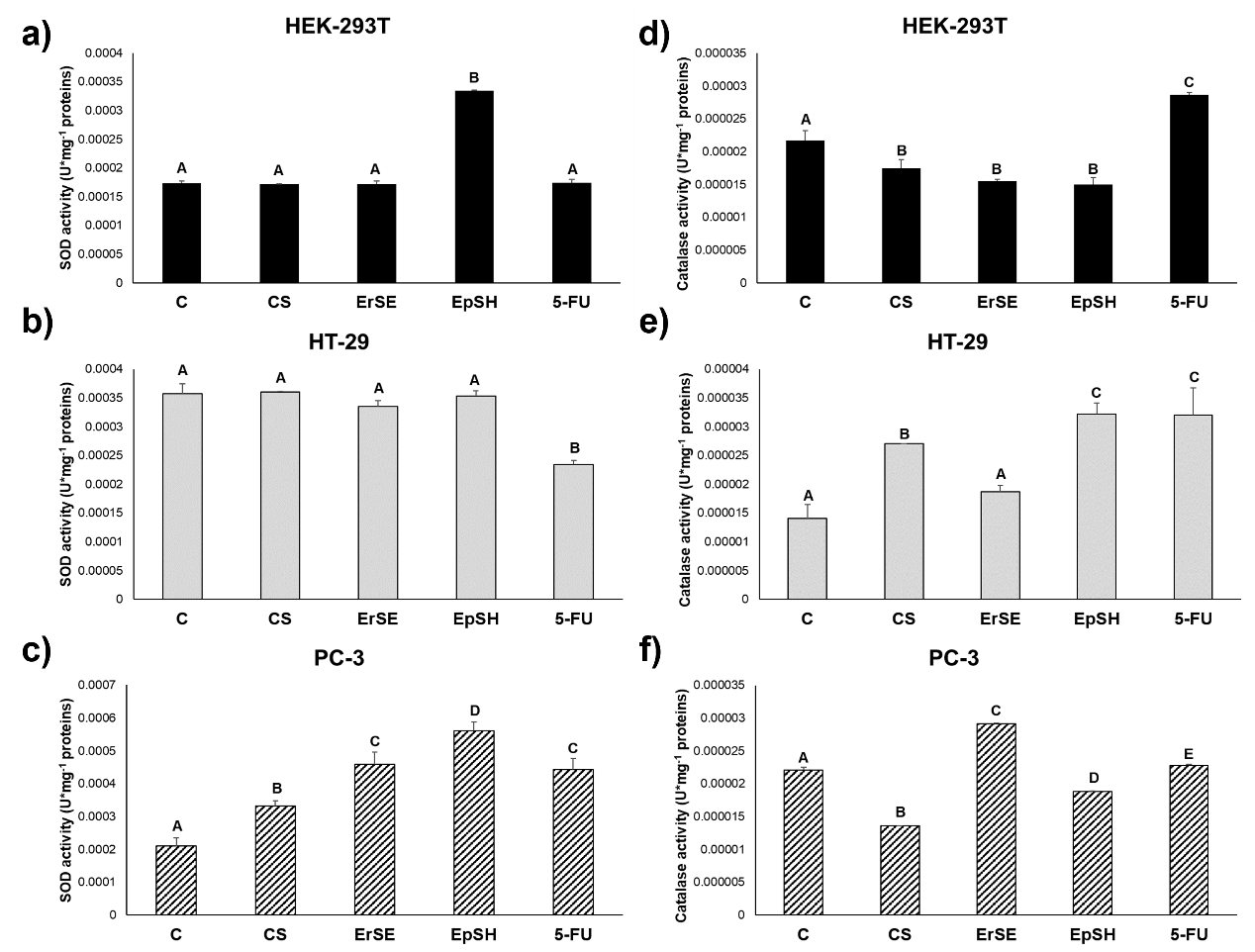


**Figure S1.** Superoxide dismutase (SOD) (a-c) and catalase (CAT) (d-f) enzymes activity evaluated on nontumor cell line (HEK-293T), colon cancer cell line (HT-29) and prostate cancer cell line (PC-3) after being exposed to the two selected extracts. Cell lines were exposed for 24 hours to a final concentration of 25 μg*mL^-1^ of each extract using 10 mL plates. All data are expressed in mean values±S.D. (n=3). A-E Different letters correspond to significant differences among treatments per cell line (p<0,05). Treatments consisted in: C= control without intervention; CS= Solvent control (0.1% ethanol); selective extracts (ethyl acetate extract from stems of *E. rubra* (ErSE); hexane extract from stems of *E. pulverulenta* (EpSH)), 5-FU= 5-Fluoro uracil (Positive control, 6.5 μg*mL^-1^, 50 μM).


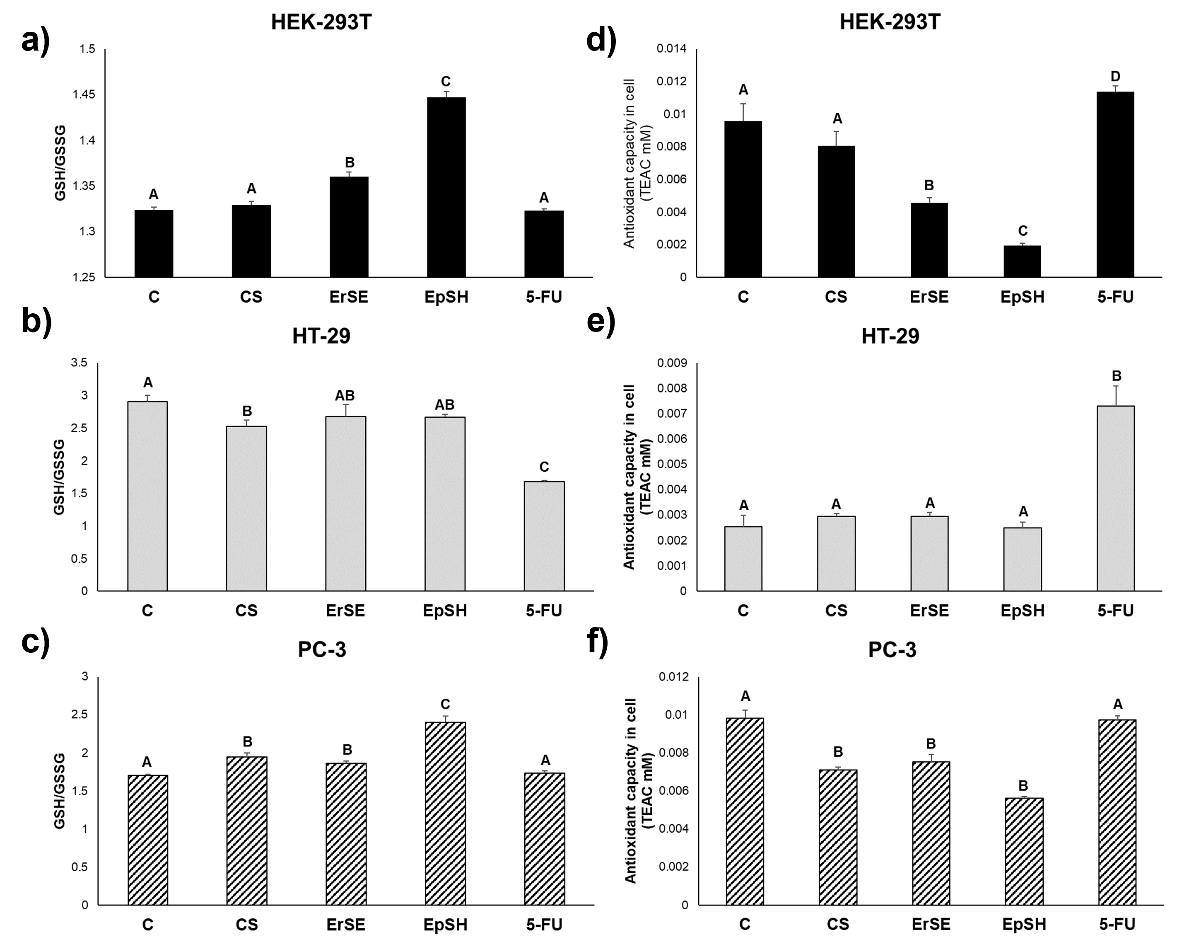


**Figure S2.** Reduced glutathione /oxidized glutathione ratio (GSH/GSSG) (a-c) and cell total antioxidant capacity (TRAPc) (d-f) evaluated on nontumor cell line (HEK-293T), colon cancer cell line (HT-29) and prostate cancer cell line (PC-3) after being exposed to the two selected extracts. Cell lines were exposed for 24 hours to a final concentration of 25 μg*mL^-1^ of each extract using 10 mL culture plates. All data are expressed in mean values±S.D. (n=3). A-D Different letters correspond to significant differences among treatments per cell line (p<0,05). Treatments consisted in: C= control without intervention; CS= Solvent control (0.1% ethanol); selective extracts (ethyl acetate extract from stems of *E. rubra* (ErSE); hexane extract from stems of *E. pulverulenta* (EpSH)), 5-FU= 5-Fluoro uracil (Positive control, 6.5 μg*mL^-1^, 50 μM).


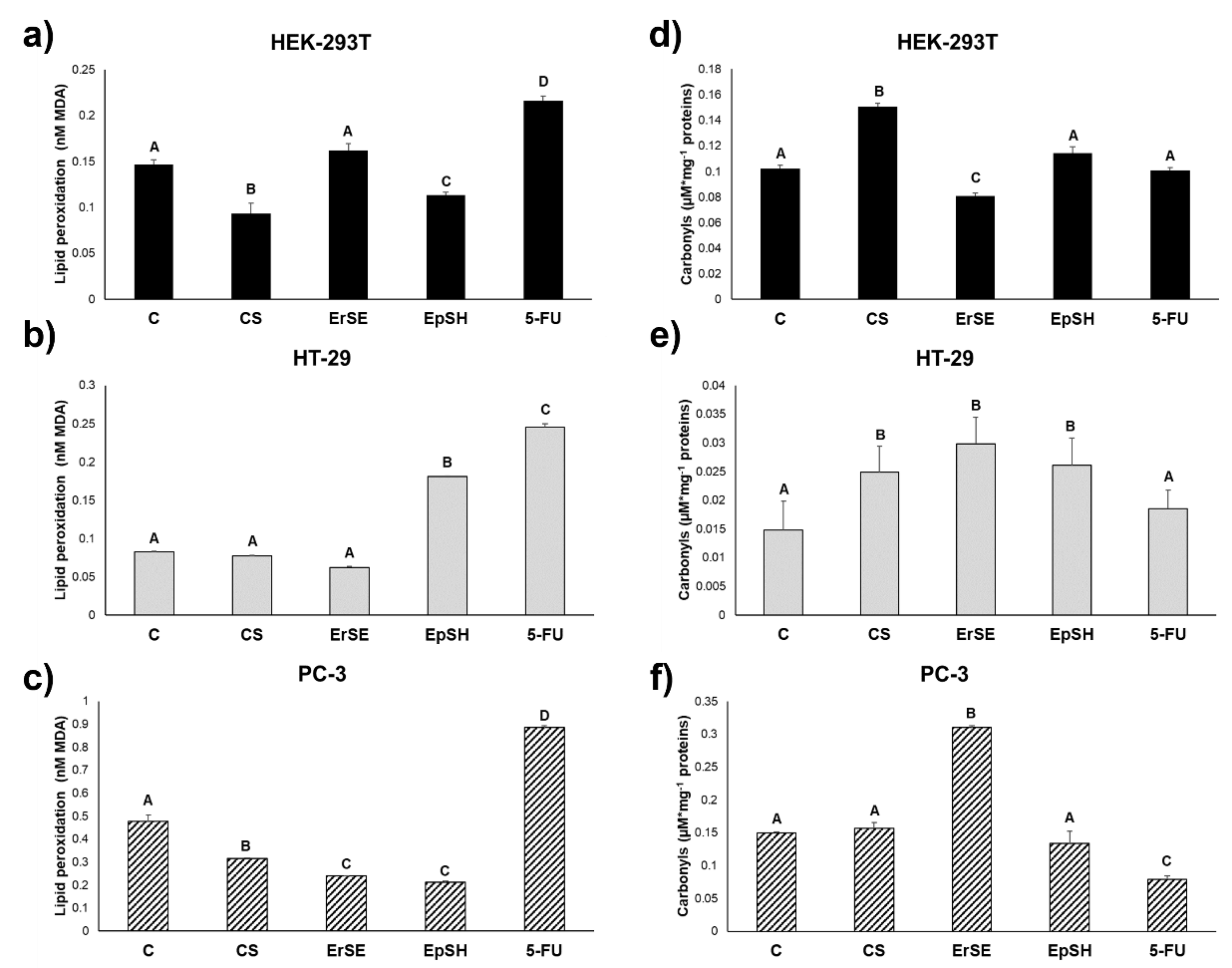


**Figure S3.** Oxidative damage measured through lipoperoxidation (a-c) and carbonyls concentration (d-f) evaluated on nontumor cell line (HEK-293T), colon cancer cell line (HT-29) and prostate cancer cell line (PC-3) after being exposed to the two selected extracts. Cell lines were exposed for 24 hours to a final concentration of 25 μg*mL^-1^ of each extract using 10 mL culture plates. All data are expressed in mean values±S.D. (n=3). A-D Different letters correspond to significant differences among treatments per cell line (p<0,05). Treatments consisted in: C= control without intervention; CS= Solvent control (0.1% ethanol); selective extracts (ethyl acetate extract from stems of *E. rubra* (ErSE); hexane extract from stems of *E. pulverulenta* (EpSH)), 5-FU= 5-Fluoro uracil (Positive control, 6.5 μg*mL^-1^, 50 μM).

1. **c) e)**

**b) d) f)**

**Figure S4.** Comparative histogram representing the number of events versus DCF fluorescence (ROS), Rho123 fluorescence (ΔΨmt) and FITC-VAD-FMK fluorescence (caspases activity) in nontumor (HEK-293T), and colon (HT-29) and prostate (PC-3) cancer cell lines after being exposed to three different concentrations (C1= 5 μg*mL^-1^, C2= 10 μg*mL^-1^, C3= 25 μg*mL^-1^) of EpSH and ErSA extracts, respectively. a) and b) Mean number of cells with DCF fluorescence after being exposed to each treatment. c) and d) Mean number of cells with Rho123 fluorescence. e) and f) Mean number of cells with FITC-VAD-FMK fluorescence. For the DCF fluorescence (ROS), 1 μM daunorubicine (DRN) as a positive control (C+), for Rho123 fluorescence (ΔΨmt), 1 μM Carbonylcyanide-p-trifluoromethoxyphenylhydrazine (FCCP) as a positive control (C+), while 1 μM daunorubicin (DRN) was used as a positive control (C+) for FITC-VAD-FMK fluorescence (caspases activity). The solvent control was 0.1% ethanol (CS) in all cases. ^A-E^ Different letters and ^*#+X^ symbols correspond to significant differences among treatments per cell line (p<0.05).

**Table S4.** GC-MS identification of EpSH (Hexane extract from *E. pulverulenta*´s steam) extract.

| **N° Peak** | **RT (Min)** | **Main Components** | **RI†** | **RI‡** | **%Area** | **Match** | **Reference** |
| --- | --- | --- | --- | --- | --- | --- | --- |
| 1 | 39.69 | Carbonic acid, eicosyl vinyl ester | 2497 | 2497 | 5.8 | 90 | [52] |
| 2 | 40.77 | 1-Tetracosanol | 2645 | 2650 | 1.21 | 88 | [53] |
| 3 | 42.27 | Tetratriacontane | 3005 | 3000 | 40.92 | 90 | [54] |
| 4 | 48.11 | Apigenin | 3120 | 3128 | 10.72 | 85 | [55] |
| 5 | 49.12 | γ-Sitosterol | 3793 | 3790 | 5.85 | 90 | [55] |
| 6 | 50.98 | 1-Heptatriacontanol | 3905 | 3941 | 5.33 | 89 | [56] |

RI†= retention indices relative to C8-C36 n-alkanes on the HP-5 MS capillary column; RI‡= retention index from the literature [ADAMS]; % Area=surface area of GC peak; Match= comparison of the mass spectra with those of the NIST 14; Co: co-elution with standard compounds available in our laboratory [57].

**Table S5.** GC-MS identification of ErSE (Ethyl acetate extract made from stems of *E. rubra*) extract.

| **N° Peak** | **RT (Min)** | **Main Components** | **RI†** | **RI‡** | **%Area** | **Match** | **Reference** |
| --- | --- | --- | --- | --- | --- | --- | --- |
| 1 | 50.89 | Lanosterol acetate | 3334 | 3339 | 99.9 | 90 | [58] |
| 2 | 51.38 | 3-Cicloursanol | 3445 | - | 0,01 | 91 | - |

RI†= retention indices relative to C8-C36 n-alkanes on the HP-5 MS capillary column; RI‡= retention index from the literature [ADAMS]; % Area=surface area of GC peak; Match= comparison of the mass spectra with those of the NIST 14; Co: co-elution with standard compounds available in our laboratory [57].
